# Resveratrol-Loaded Hydrogel Contact Lenses with Antioxidant and Antibiofilm Performance

**DOI:** 10.3390/pharmaceutics13040532

**Published:** 2021-04-11

**Authors:** María Vivero-Lopez, Andrea Muras, Diana Silva, Ana Paula Serro, Ana Otero, Angel Concheiro, Carmen Alvarez-Lorenzo

**Affiliations:** 1 Departamento de Farmacología, Farmacia y Tecnología Farmacéutica, I+D Farma (GI-1645), Facultad de Farmacia and Health Research Institute of Santiago de Compostela (IDIS), Universidade de Santiago de Compostela, 15782 Santiago de Compostela, Spain; mariavivero.lopez@usc.es; 2 Departamento de Microbiología, Facultad de Biología, Edificio CIBUS, Universidade de Santiago de Compostela, 15782 Santiago de Compostela, Spain; andrea.muras@usc.es (A.M.); anamaria.otero@usc.es (A.O.); 3 Centro de Química Estrutural, Instituto Superior Técnico, Universidade de Lisboa, Av. Rovisco Pais, 1049-001 Lisboa, Portugal; dianacristinasilva@tecnico.ulisboa.pt (D.S.); anapaula.serro@tecnico.ulisboa.pt (A.P.S.); 4 CIIEM, Instituto Superior de Ciências da Saúde Egas Moniz, Campus Universitário, Quinta da Granja, Monte de Caparica, 2829-511 Caparica, Portugal

**Keywords:** antibiofilm, antioxidant, drug-eluting contact lenses, microbial keratitis, endophthalmitis, device-related ocular infections

## Abstract

Contact lenses (CLs) are prone to biofilm formation, which may cause severe ocular infections. Since the use of antibiotics is associated with resistance concerns, here, two alternative strategies were evaluated to endow CLs with antibiofilm features: copolymerization with the antifouling monomer 2-methacryloyloxyethyl phosphorylcholine (MPC) and loading of the antioxidant resveratrol with known antibacterial activity. MPC has, so far, been used to increase water retention on the CL surface (Proclear^®^ 1 day CLs). Both poly(hydroxyethyl methacrylate) (HEMA) and silicone hydrogels were prepared with MPC covering a wide range of concentrations (from 0 to 101 mM). All hydrogels showed physical properties adequate for CLs and successfully passed the hen’s egg-chorioallantoic membrane (HET-CAM) test. Silicone hydrogels had stronger affinity for resveratrol, with higher loading and a slower release rate. Ex vivo cornea and sclera permeability tests revealed that resveratrol released from the hydrogels readily accumulated in both tissues but did not cross through. The antibiofilm tests against *Pseudomonas aeruginosa* and *Staphylococcus aureus* evidenced that, in general, resveratrol decreased biofilm formation, which correlated with its concentration-dependent antibacterial capability. Preferential adsorption of lysozyme, compared to albumin, might also contribute to the antimicrobial activity. In addition, importantly, the loading of resveratrol in the hydrogels preserved the antioxidant activity, even against photodegradation. Overall, the designed hydrogels can host therapeutically relevant amounts of resveratrol to be sustainedly released on the eye, providing antibiofilm and antioxidant performance.

## 1. Introduction

The surfaces of contact lenses (CLs) and intraocular lenses (IOLs) are quite prone to the formation of bacterial biofilms, which may cause severe infections in the ocular structures [1,2]. Although CL materials have undergone a profound evolution over the last few years, the incidence of microbial-related ocular diseases has not decreased [3]. Approximately 4.2 out of 10,000 CL wearers suffer from microbial keratitis, mainly caused by bacteria (>90%) [4]. CL wearing continues to be the most relevant risk factor for the development of microbial keratitis, although not all CLs are the same in terms of being prone to bacterial growth [1,2,5,6]. The risk increases in the following order: daily wear rigid gas permeable CLs < daily wear soft CLs < extended (overnight) wear CLs [1,5,7]. Bacteria can come into contact with the eye through the fingers when inserting and removing the lens or through the CL itself if the care solutions or storage cases are contaminated [1]. Once the CL is inserted into the eye, proteins and other tear components adsorb onto its surface, which facilitates the adhesion of bacteria. Bacterial biofilms grown on CLs play a crucial role in keratitis disease, being generally associated with the Gram-negative *Pseudomonas aeruginosa* and the Gram-positive *Staphylococcus aureus* [1,2,3,6]. Ocular biofilms can remain on the CL surface for long periods of time, regularly releasing planktonic cells and bacterial products such as endotoxins that can damage the corneal epithelium and induce other ophthalmic diseases, such as dry eye [2,8]. CLs do not only support biofilm formation but also interfere with the normal host defense mechanisms, such as tearing and blinking, and modify the corneal epithelium, facilitating the adhesion and colonization by opportunistic bacteria [2,5,7]. Once bacteria adhere to the injured corneal epithelium, microbial keratitis can progress [1].

Biofilm formation is also a problem for the safety of IOLs. After placement of an IOL in cataract surgery, patients may develop endophthalmitis, a serious form of intraocular inflammation caused by the introduction of a microbial pathogen in the posterior segment of the eye [5,9]. The process starts with the contamination of IOLs with bacteria that can be present in the conjunctiva (e.g., *S. epidermidis*, *S. aureus* or *P. aeruginosa*) during insertion and their subsequent transport from the ocular surface to the posterior chamber. The relatively static environment of vitreous humor favors the development of the biofilm and the associated infection [5]. Overall, although CLs and IOLs have shown great success in improving vision problems, the incidence of eye infections associated with these devices represents a significant concern regarding their use. Hence, the development of new lenses with antibiofilm substances in their composition is receiving increasing attention [3,10].

**Figure 1:**
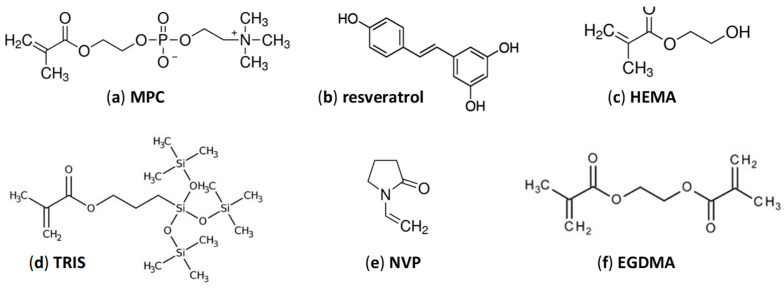
Chemical structure of (**a**) 2-methacryloyloxyethyl phosphorylcholine (MPC), (**b**) *trans*-resveratrol, (**c**) 2-hydroxyethyl methacrylate (HEMA), (**d**) 3-(methacryloyloxy)propyltris(trimethylsiloxy)silane (TRIS), (**e**) *N*-vinylpyrrolidone (NVP) and (**f**) ethylene glycol dimethacrylate (EGDMA).

Consequently, 2-Methacryloyloxyethyl phosphorylcholine (MPC) (Figure 1) has been explored to endow polymeric networks with improved biocompatibility and protein-resistant surfaces [11,12,13,14,15,16]. MPC is highly hydrophilic and its bioinspired phosphorylcholine group resembles the phospholipid headgroups present in the cell membrane. In this regard, CLs made of copolymers of 2-hydroxyethyl methacrylate (HEMA) and MPC, commercially available as Proclear^®^, claim to remain wet for prolonged periods of time and thus to provide improved comfort to the wearers [17]. Surface grafting of MPC has been shown to enhance the ocular tolerance of hydrophobic acrylic IOLs [13] and silicone IOLs [18]. Furthermore, antifouling coatings based on MPC may provide biomaterials with higher wettability and lower protein adsorption [19] and have been shown to be useful to prevent the adhesion of bacteria due to the formation of highly hydrated, flexible interfaces that avoid the deposition of conditioning substances [20].

In parallel to the design of surfaces that may prevent the adhesion of bacteria, the incorporation into the lenses of natural compounds that can act as biofilm inhibitors is gaining attention. In this regard, resveratrol (trans-3,5,4′-trihydroxystilbene) (Figure 1), which is a natural antioxidant polyphenol [21] with good ocular tolerance [22,23], has been reported to interfere in vitro with the bacterial communication process known as quorum sensing (QS) and to inhibit biofilm formation by clinically relevant bacteria such as *S. aureus*, *P. aeruginosa* and *Vibrio cholerae* [24,25,26,27]. The interference in QS processes may increase the susceptibility to antibiotics and downregulate natural, industrial and clinical biofilms [28,29]. Resveratrol is also believed to cause bacterial growth inhibition due to a combination of mechanisms involving membrane damage and inhibition of ATP synthase and efflux pumps, which facilitates the intracellular accumulation of other antimicrobial substances [30,31]. The minimum inhibitory concentration (MIC) of resveratrol for the Gram-positive *S. aureus*, *Streptococcus pyogenes* and *Enterococcus faecalis* was reported to be between 100 and 200 µg/mL; meanwhile, Gram-negative bacteria such as *P. aeruginosa*, *E. coli* or *Klebsiella pneumoniae* showed lower susceptibility to resveratrol [32].

This work relies on the hypothesis that the design of hydrogels containing MPC moieties and incorporating resveratrol may exhibit a synergism in terms of the prevention of biofilm formation on CLs, while resveratrol may also lead to other beneficial effects related to its antioxidant capability. Some eye diseases caused by oxidative stress mechanisms, such as age-related macular degeneration, cataracts and glaucoma, may be treated or prevented with antioxidants [23,33]. To the best of our knowledge, loading of resveratrol in CLs has not been tested before. Thus, the aim of this work was to prepare hydrogel CLs and silicone hydrogel CLs containing MPC and loaded with resveratrol to decrease the incidence of ocular infections and manage some eye diseases caused by oxidative stress mechanisms. To carry out the work, hydrogels were prepared covering a wide range of MPC ratios and then the loading and release profiles of resveratrol were evaluated. An extensive characterization of the materials, comprising the evaluation of their protein adsorption and antibiofilm and antioxidant capabilities, was performed. Commercially available Proclear^®^ 1 day CLs were used as a control for comparison purposes.

## 2. Materials and Methods

### 2.1. Materials

Firstly, 3-(Methacryloyloxy)propyltris(trimethylsiloxy)silane (TRIS) was from Alfa Aesar by Thermo Fisher (Thermo Fisher, Kandel, Germany); 2-Hydroxyethyl methacrylate (HEMA), (3-(4,5-dimethylthiazol-2-yl)-2,5-diphenyltetrazolium bromide) (MTT), calcium chloride dihydrate (CaCl_2_·2H_2_O) and sodium dihydrogen phosphate monohydrate (NaH_2_PO_4_·H_2_O) were from Merck KGaA (Merck KGaA, Darmstadt, Germany); 2-Methacryloyloxyethyl phosphorylcholine (MPC), 1-vinyl-2-pyrrolidinone, ethylene glycol dimethacrylate (EGDMA), dichlorodimethylsilane, 2,2′-azobis(2-methylpropionitrile) (AIBN), 2,2-diphenyl-1-picrylhydrazyl and lysozyme from chicken egg white were from Sigma-Aldrich (Sigma-Aldrich, St. Louis, MO, USA). Resveratrol was from ChemCruz, Santa Cruz Biotechnology Inc. (Santa Cruz Biotechnology Inc., Dallas, TX, USA). Albumin bovine fraction V standard grade (pH 7) was from SERVA Electrophoresis GmbH (SERVA Electrophoresis GmbH, Heidelberg, Germany). Di-sodium hydrogen phosphate anhydrous (N**a_2_**HPO_4_), magnesium chloride 6-hydrate (MgCl_2_·6H_2_O) and potassium di-hydrogen phosphate (KH_2_PO_4_) were from PanReac Química S.L.U. (Química S.L.U., Barcelona, Spain). Sodium bicarbonate (NaHCO_3_) was from Probus S.A. (Probus S.A., Barcelona, Spain). Potassium chloride (KCl), sodium chloride (NaCl) and calcium chloride anhydrous (CaCl_2_) were from Scharlab S.L. (Scharlab S.L., Barcelona, Spain). Ethanol absolute 99.9% and sodium hydroxide (NaOH) were from VWR Chemicals (VWR Chemicals, Fontenary-Sous-Bois, France). Bacto™ tryptone and bacto™ yeast extract were from Becton, Dickinson and Company (Becton, Dickinson and Company, Le Pont de Claix, France) and tryptic soy broth (TSB) was from Oxoid S.A. (Oxoid S.A., Madrid, Spain). Ultrapure water (resistivity >18.2 MΩ·cm) was obtained by reverse osmosis (MilliQ^®^, Millipore Ibérica, Madrid, Spain). Phosphate-buffered saline (PBS) medium was prepared with 8 g of NaCl, 0.3 g of KCl, 0.73 g of N**a_2_**HPO_4_ and 0.2 g of KH_2_PO_4_ for 1 L with pH 6.5. Carbonate buffer was prepared with 1.24 g of NaCl, 0.071 g of KCl, 0.02 g of NaH_2_PO_4_·H_2_O, 0.49 g of NaHCO_3_, 0.023 g of CaCl_2_ and 0.031 g of MgCl_2_·6H_2_O for 200 mL with pH 7.2. Simulated lachrymal fluid (SLF) was prepared with the following composition: 6.78 g/L NaCl, 2.18 g/L NaHCO_3_, 1.38 g/L KCl and 0.084 g/L CaCl_2_·2H_2_O with pH 7.5 [34]. Proclear^®^ 1 day CLs (Omafilcon A, CooperVision^®^, Lake Forest, CA, USA), diopter −3.00, water content 60%, Dk/t 28, were acquired from a local optical store.

### 2.2. Hydrogel Synthesis

Different monomer solutions (Table 1) were prepared at room temperature under magnetic stirring (400 rpm). EGDMA was used as crosslinker and AIBN as initiator. The monomer solutions were injected (25 G needle) into molds made of presilanized glass plates (12 × 14 cm) fixed with 0.30 mm Teflon frame (silicone-hydrogels) or 0.45 mm silicone frame (HEMA-hydrogels). The polymerization was carried out at 50 °C for 12 h and at 70 °C for other 24 h.

After polymerization, hydrogel sheets were demolded by injecting a small amount of water into the molds with a syringe, washed in 1 L of boiling distilled water for 15 min to remove unreacted monomers and cut with punches into different sizes according to the needs of each test. Hydrogel pieces were washed in MilliQ^®^ water (1 L) at room temperature, replacing the medium two or three times per day until the complete removal of unreacted monomers, which was confirmed by measuring the absorbance of aliquots of the washing medium (UV–Vis spectrophotometer Agilent 8453, Waldbronn, Germany), and dried at 70 °C for 24 h. Proclear^®^ 1 day CLs were washed using the same procedure and dried at 40 °C for 2 h and 70 °C for a further 2 h. Some hydrogel pieces were directly freeze-dried and scanning electron microscopy (SEM) images of their surfaces were recorded (FESEM Zeiss Ultra Plus, Oberkochen, Germany).

### 2.3. Water Uptake

Dry Proclear^®^ 1 day CLs and discs of each hydrogel (10 mm diameter) were weighed and placed into Falcon^®^ tubes with 5 mL of water, SLF or resveratrol solution (100 µg/mL in ethanol:water 10:90 *v*/*v*) at room temperature. Three replicates were tested. At preset times (each hour during the first 8 h and then every 24 h for 5 days), the discs were weighed after carefully wiping their surfaces with absorbent paper to remove excess water. The increase in weight was recorded to calculate the water uptake as a percentage using the following Equation (1).

(1) $$\mathrm{Water}\;\mathrm{uptake}\;\left(\%\right)=\;\frac{W_{t}-W_{0}}{W_{0}}\times 100$$

In this equation, W_0_ and W_t_ represent the weight of dry hydrogel and swollen hydrogel at time t, respectively.

### 2.4. Transmittance

The light transmittance (%) of swollen hydrogel discs and Proclear^®^ 1 day CLs was recorded using a UV–Vis spectrophotometer (Agilent Cary 60 UV-Vis, Waldbronn, Germany) from 200 to 700 nm with 1 nm intervals. All the measurements were carried out at least in triplicate after swelling in water, SLF and resveratrol solution (100 µg/mL in ethanol:water 10:90 *v*/*v*).

### 2.5. Wettability

The wettability of the hydrogels was determined by the captive bubble method, following a methodology described previously [35]. Hydrogel discs (10 mm in diameter) were hydrated in 5 mL of water for at least 24 h. Then, the discs were placed horizontally in a measuring cell filled with water. Air bubbles (3–4 µL) were formed and released underneath the inferior surface of the discs, using a micrometer syringe with an end-curved needle. The bubbles adhered to the hydrogel surface and the water contact angle was measured as the angle formed between the hydrogel surface and the tangent to the bubble at the triple point where water/air/hydrogel coexists (Figure S1, Supplementary Information). Images were taken at set time intervals for 1 min using a video camera (JAI CV-A50, Copenhagen, Denmark) mounted on an optical microscope (Wild M3Z, Leica Microsystems, Jena, Germany) and connected to a frame grabber (Data Translation DT3155, Measuring Computing Corp., Norton, MA, USA). The acquisition and analysis of the images were performed using the ADSA-P software (Axisymmetric Drop Shape Analysis Profile; Applied Surface Thermodynamics Research Associates, Toronto, Canada). Three discs were used for each formulation, and eight to ten bubbles were created for each disc; in total, there were 30 bubbles per formulation.

### 2.6. Mechanical Properties

HEMA- and silicone-hydrogel-hydrated (in water) strips (16 × 9 mm) were fixed at room temperature to the upper and lower clamps (gap 7 mm) of a TA.XT Plus Texture Analyzer (Stable Micro Systems, Ltd., Surrey, UK) fitted with a 5 Kg load cell. Stress–strain plots were recorded at a crosshead speed of 0.1 mm·s^−1^ at least in triplicate. Young’s modulus was calculated from the slope of the linear portion of the stress versus strain curves. The Young’s modulus of Proclear^®^ 1 day CLs was also evaluated.

### 2.7. Resveratrol Loading and Release Tests

HEMA-hydrogel-dried discs (37 °C, 24 h) with 10 mm diameter (average weight 39 mg), silicone-hydrogel-dried discs (37 °C, 24 h) with 10 mm diameter (average weight 13 mg) and Proclear^®^ 1 day dried CLs (37 °C, 24 h) with 14.2 mm diameter (average weight 13 mg) were placed, in separate Falcon^®^ tubes with 7 mL of resveratrol in ethanol:water 10:90 *v*/*v* solution (100 µg/mL). Then, they were placed in an Incubating Mini Shaker (VWR) at 36 °C and 180 rpm protected from light. The test was carried out in quadruplicate.

The absorbance of the loading solution was monitored at 305 nm (UV–Vis spectrophotometer Agilent 8534, Waldbronn, Germany) by taking aliquots of 250 µL for the first 8 h and aliquots of 500 µL for subsequent measurements (the liquid removed was not replaced with fresh solution). The aliquots were leveled to 5 mL with ethanol:water 10:90 *v*/*v* before absorbance measurement. The amount of resveratrol loaded was calculated from the difference between the initial and the final amount of drug in the solution using a validated calibration curve obtained in ethanol:water 10:90 *v*/*v* and considering the amount of resveratrol lost in the monitoring. A loading test was also carried out for 72 h without intermediate measurements. The network/water partition coefficient (K_N/W_) was calculated as the difference between the total amount loaded and the amount that could be hosted in the aqueous phase (using the water uptake values) and divided by the concentration of resveratrol in the loading solution [34].

Release experiments were carried out by placing resveratrol-loaded discs (previously rinsed with NaCl 0.9%) in Falcon^®^ tubes with 6 mL of NaCl 0.9%. The tubes were kept in an Incubating Mini Shaker (VWR) at 36 °C and 180 rpm protected from light. The test was carried out in quadruplicate. Samples of the medium were taken every hour in the first 8 h of the experiment, and the absorbance was measured at 305 nm using a UV–Vis spectrophotometer (Agilent 8534, Waldbronn, Germany). The samples were immediately returned to the corresponding vial, except for HEMA hydrogels and Proclear^®^ 1 day CLs. For these hydrogels, aliquots (1 mL) of the release medium sampled in the 3 to 8 h interval were replaced with the same volume of NaCl 0.9% fresh solution. Once the 8 h time point samples were measured, the release medium in all tubes was increased to 12 mL with the addition of 6 mL of NaCl 0.9% fresh solution. Therefore, from this time point (8 h) until 25 h was reached, the volume of the release medium was 12 mL. At 25 h, HEMA hydrogels and Proclear^®^ 1 day CLs were transferred to new Falcon^®^ tubes containing 12 mL of NaCl 0.9% fresh solution, and the test proceeded for one month. Contrastingly, silicone hydrogels remained in the initial Falcon^®^ tubes since the concentration of resveratrol achieved was quite low. Resveratrol concentration values were calculated from the absorbance at 305 nm using a calibration curve of resveratrol dissolved in NaCl 0.9% solution. The amounts removed and the corresponding dilution of the sample, if needed, were considered to calculate the total amount released.

### 2.8. Resveratrol Stability

Since *trans*-resveratrol is highly sensitive to certain light conditions [36], stability during storage under dark and once exposed to various light conditions was investigated. First, solutions of resveratrol in ethanol:water 10:90 *v*/*v* (5 µg/mL) and NaCl 0.9% (6 µg/mL) were placed into Falcon^®^ tubes (36 °C, 180 rpm) for 24 h and 20 days, respectively, protected from light, and the UV–Vis spectra recorded at preset times (UV–Vis spectrophotometer Agilent 8534, Waldbronn, Germany). The stability of resveratrol in ethanol:water 10:90 *v*/*v* (5 µg/mL) against white light (HITACHI 8 W F8T5 daylight, Chiyoda, Japan) (14 cm gap from the lamp) and the light in the working area of the laboratory was also evaluated for 24 h by monitoring the UV–Vis spectrum. To evaluate the capacity of the hydrogels to protect resveratrol from light degradation, resveratrol-loaded HEMA- and silicone-based hydrogels (soaked in a 100 µg/mL resveratrol in ethanol:water 10:90 *v*/*v* solution for 72 h) were placed into empty quartz cells and exposed to white light (HITACHI 8 W F8T5 daylight, Chiyoda, Japan) for 3 h (14 cm gap from the lamp) at room temperature. The amount of resveratrol loaded and released was monitored, following the same procedure as described above.

### 2.9. Ex Vivo Corneal and Scleral Permeability Tests

Resveratrol corneal and scleral permeability tests were carried out, in triplicate, according to a previously described protocol [37] for selected silicone- and HEMA-based hydrogels. The assay was also carried out for resveratrol-loaded Proclear^®^ 1 day CLs and a resveratrol solution (1 mL, 70 µg/mL in ethanol:water 10:90 *v*/*v*) prepared considering the maximum amount that the hydrogels could release in 6 h (estimated from release tests described in Section 2.7). Fresh porcine eyes from a local slaughterhouse were transported immersed in PBS in an iced bath. Then, intact scleras and corneas with 2–3 mm of surrounding sclera were isolated with the help of a scalpel and tongs, washed with PBS and fitted into vertical diffusion Franz cells. Donor and receptor chambers were filled with carbonate buffer with a pH of 7.2. The receptor medium (6 mL) was kept at 37 °C under gentle magnetic stirring (400 rpm). After 30 min equilibration, the buffer of the donor chambers was removed and replaced with resveratrol-loaded discs (soaked in 100 µg/mL resveratrol in ethanol:water 10:90 *v*/*v* solution for 72 h at 36 °C and 180 rpm) immersed in 2 mL of NaCl 0.9%. The area available for permeation was 0.785 cm^2^. The donor chambers were covered with parafilm to prevent evaporation and protected from light to avoid resveratrol degradation. At 30 min and then each hour for 6 h, 1 mL of sample was taken from the receptor chamber and replaced with the same volume of carbonate buffer with a pH of 7.2, taking care to remove any bubbles from the diffusion cells.

The amount of resveratrol permeated was quantified using a JASCO (Tokyo, Japan) HPLC (AS-4140 Autosampler, PU-4180 Pump, LC-NetII/ADC Interface Box, CO-4060 Column Oven, MD-4010 Photodiode Array Detector), fitted with a C18 column (Waters Symmetry C18, 5 µm, 4.6 × 250 mm) and operated with ChromNAV software (ver. 2.2.8.5, JASCO, Tokyo, Japan). The analysis was done by isocratic elution with a mobile phase of water:methanol (50:50) at 1 mL/min, at 35 °C and with a run time of 8 min. The injection volume was 50 µL and the UV detector was set at 305 nm. Retention time was 4.6 min. The method was validated using two different calibration curves of resveratrol in methanol:water 50:50 *v*/*v*, one in the 0.05–2 µg/mL range and other in the 1–6 µg/mL range. The detection and quantification limits were calculated from the first calibration curve to be 0.007 and 0.016 µg/mL, respectively. This low-range calibration curve included many points for the precise and accurate quantification of resveratrol in the diluted samples.

After 6 h of assay, aliquots of the donor chambers were collected to quantify the amount of resveratrol remnant. The corneas and scleras were visually inspected to verify that none of them had cracks or modifications and then placed in Falcon^®^ tubes with 3 mL of ethanol:water (50:50 *v*/*v*) medium at 37 °C under agitation. After 24 h, they were sonicated for 99 min at 37 °C, centrifuged (1000 rpm, 5 min, 25 °C), and the supernatant was filtered (Scharlau^®^ Syringe Filter, 0.22 µm 13 mm PTFE hydrophilic), centrifuged again (14,000 rpm, 20 min, 25 °C) and filtered again to be analyzed by HPLC, as described above.

### 2.10. HET-CAM Test

The Hen’s Egg Test on the Chorioallantoic Membrane (HET-CAM) assay was carried out using fertilized hens’ eggs (50–60 g, Coren, Spain) after incubation in a climatic chamber (Ineltec CC SR 0150, Barcelona, Spain), as previously described [38]. On the ninth day, a circular cut of 1 cm in diameter was made on the wider extreme to remove the eggshell. The inner membrane was wetted and removed, and resveratrol-loaded hydrogel discs (as explained above) were placed on the CAM. Solutions of 0.1 N NaOH and 0.9% NaCl (300 µL) were used as positive and negative control, respectively. The vessels of CAM were observed for 5 min and the time at which hemorrhage (vessels bleeding), vascular lysis (vessels disintegration) or coagulation (denaturalization of intra and extravascular proteins) appeared was recorded. The irritation score (IS) was calculated as previously reported [39].

### 2.11. Protein Adsorption

A quartz crystal microbalance with dissipation (QCM-D, E4 from Q-Sense, Gothenburg, Sweden) was used to study the adsorption of albumin and lysozyme onto selected silicone- and HEMA-based hydrogels (Table 1). As previously described [40], gold-coated quartz crystals (5 MHz) were treated with UV–ozone for 15 min, rinsed with water and dried with nitrogen. The crystals were coated with a layer of polystyrene (20 µL, 2% wt in toluene) by spin coating (2000 rpm, 30 s) and 20 µL of the correspondent silicone–monomer mixture was deposited over this layer by spin coating (5000 rpm, 30 s). The polystyrene film was only applied for silicone-based mixtures since the direct adhesion of these onto the gold surface was poor. For HEMA-based mixtures, the deposition could be done directly on the gold-coated quartz crystals. In both cases, the polymerization was then carried out at 50 °C for 30 min and 70 °C for 1 h [41].

The crystals were mounted on the QCM-D cells and the experimental baselines were obtained with the hydrogel films pre-hydrated in SLF. Normalized frequency (∆f/n, where *n* corresponds to the number of the harmonic) and dissipation (∆D) changes for the 1st, 3rd, 5th, 7th, 9th and 11th harmonics were registered throughout the experiments. Protein solutions (albumin 0.05 mg/mL in SLF and lysozyme 1.9 mg/mL in SLF) were added and remained for approximately 2.5 h in contact with the crystals. A final rinsing was done with SLF and the monitored signals were left to stabilize for 20 min. The experiments were carried out in quadruplicate at 36 °C. After each assay, the crystals were retrieved by dipping for 5 s in piranha solution (H_2_SO_4_:H_2_O_2_, 7:3 *v*/*v*), followed by washing in a 2% (*v*/*v*) Hellmanex solution and washing two times in DD water under ultrasound for 15 min each. Finally, the crystals were dried using nitrogen flux and stored.

### 2.12. Antibiofilm Properties

Silicone and HEMA hydrogels (1, 4, 5 and 6) and Proclear^®^ 1 day CLs were tested in triplicate for 6 h of growth against P. aeruginosa and 48 h of growth against S. aureus. Bacterial biofilms were grown on hydrated hydrogel pieces immersed in culture medium using a modified Amsterdam Active Attachment (AAA) model [42] assembled with the tested materials (Figure S2 in Supporting Information). Both non-loaded and resveratrol-loaded hydrogels were tested. As controls, bacteria growth on glass coverslips was monitored both in the absence and in the presence of resveratrol in the culture medium. For this, resveratrol solutions in ethanol:water 10:90 *v*/*v* previously filtered (Biofil^®^ Syringe Filter, 0.22 µm PES membrane) were added to Luria–Bertani Broth (LB) for P. aeruginosa (4, 12 and 250 µg/mL resveratrol final concentration) and Tryptic Soy Broth (TSB-1) for S. aureus (5, 17 and 250 µg/mL resveratrol final concentration). In any case, the growth medium was diluted less than 5% with the resveratrol solution.

#### 2.12.1. Bacterial Strains and Growth Conditions

*S. aureus* ATCC25923 (ATCC, Manassas, VA, USA) and *P. aeruginosa* PAO1 (Lausanne sub-line, donated by M. Cámara, Univ. of Nottingham, Nottingham, UK) biofilm-forming bacteria were routinely cultured at 37 °C in TSB-1 and LB, respectively. TSB-1 (15 g of TSB and 2.5 g of NaCl for 500 mL) and LB (5 g of tryptone, 2.5 g of yeast extract and 5 g of NaCl for 500 mL) media were prepared in distilled water. Both culture media were magnetically stirred at 200 rpm until complete dissolution and then autoclaved (121 °C, 1 atm, 15 min) to avoid contamination.

#### 2.12.2. Pre-Inocula and Inocula Preparation

Pre-inocula were prepared by inoculating sterile Erlenmeyer flasks containing culture medium (10 mL) with a colony of a 24 h plate of the corresponding biofilm-forming bacterial pathogen. The flasks were incubated at 37 °C for 12 h (*P. aeruginosa*) or 24 h (*S. aureus*) at 100 rpm. For inocula preparation, the optical density of the pre-inocula was measured after incubation at 600 nm (UV–Vis spectrophotometer Thermo Scientific Helios Omega) and adjusted to 0.05 (*S. aureus*) or 0.01 (*P. aeruginosa*) by dilution with the corresponding culture medium in sterile Falcon^®^ tubes of 50 mL. The pre-inocula were diluted around 400 and 100-fold for *P. aeruginosa* and *S. aureus*, respectively. Finally, the inocula were gently homogenized and divided among 12-well cell culture plates (4 mL per well). All procedures were performed in a biological safety cabinet.

#### 2.12.3. Amsterdam Active Attachment (AAA) Model Preparation and Pncubation

The hydrogels were loaded with resveratrol (as explained above), with the only difference being that they were autoclaved (121 °C, 1 atm, 15 min) and dried for 72 h at 37 °C before soaking in the resveratrol solution (7 mL, 37 °C, 100 rpm). The amount loaded was estimated from the difference between the initial and final amount of resveratrol in the solution, calculated from absorbance measurements performed at 305 nm (as above).

All loaded hydrogel pieces were carefully placed in the silicone supports of special metallic covers previously autoclaved (121 °C, 1 atm, 15 min), using a scalpel and tongs, in a biological safety cabinet. Then, the setup was immersed in the 12-well cell culture plates containing 4 mL of the corresponding culture media and bacteria.

All hydrogels were tested in triplicate and incubated at 37 °C under static conditions for 6 h and 48 h for *P. aeruginosa* and *S. aureus*, respectively. The *S. aureus* medium was changed every 12 h by moving the models to new cell culture plates previously filled with 4 mL of fresh TSB-1 per well. In all cases, after each media exchange period, the absorbance of the culture medium was measured at 600 nm (UV–Vis spectrophotometer Thermo Scientific Helios Omega), to evaluate planktonic bacterial growth. A similar protocol was used to monitor bacteria growth onto glass coverslips when the medium was supplemented with different concentrations of resveratrol.

#### 2.12.4. Biofilm Susceptibility

After the incubation period at 37 °C, the viability of the bacterial biofilms was evaluated using a modified MTT assay [43]. Hydrogel pieces were individually placed in sterile tubes containing 3.6 mL of PBS and then sonicated for 15 min to separate and homogenize the biofilms. Then, MTT solution (5 mg/mL, 400 µL) was added to each tube and the tubes were incubated at 37 °C for 30 min. Half of the MTT-containing PBS medium was removed and replaced with acid isopropanol (5% (*v*/*v*) 1M HCl in isopropanol). After 5 s vortexing, aliquots of the medium (1 mL) were taken and their absorbance measured at 570 nm (UV–Vis spectrophotometer Thermo Scientific Helios omega). PBS medium treated in the same way was used as blank.

### 2.13. Antioxidant Properties

The antioxidant activity of resveratrol released from Proclear^®^ 1 day CLs, HEMA and silicone hydrogels was determined using a modified DPPH assay [44]. The antioxidant activity was proportional to the disappearance of radical 2,2-diphenyl-1-picrylhydrazyl (DPPH•) in the samples by accepting hydrogen from resveratrol, with the corresponding change in color from purple to yellow and decrease in absorption at 517 nm [45]. HEMA- and silicone-hydrogel-dried discs were loaded with resveratrol (as above) and, then, the release was carried out, as described previously, for 12 and 24 h. The antioxidant activities of the loading solution (100 µg/mL in ethanol:water 10:90 *v*/*v* medium) and the freshly prepared release medium (NaCl 0.9%) were also quantified. Non-loaded hydrogels in NaCl 0.9% were used as controls to confirm that there were no leaching substances that could cause false antioxidant activity during the test. To carry out the test, a 0.1 mM solution of DPPH• in ethanol was freshly prepared and stored in a flask protected from light. Then, an aliquot of each release medium (1 mL) was mixed with 1 mL of DPPH• solution and vortexed for 5 s. After 30 min of incubation in the dark, the absorbance was measured at 517 nm (UV–Vis spectrophotometer Agilent 8534, Waldbronn, Germany). The test was carried out at least in triplicate. The DPPH• scavenging capacity was expressed as µg/mL of DPPH in the reaction medium and calculated from a validated calibration curve of DPPH• in ethanol (4–25 µg/mL). The DPPH• scavenging effect (%) was obtained using the following Equation (2), where *A_C_* is the absorbance at 517 nm of the control and A_S_ is the absorbance of the test compound.

(2) $$\mathrm{DPPH}\;\mathrm{scavenging}\;\mathrm{effect}\;\left(\%\right)=\left(1-\frac{A_{s}}{A_{c}}\right)\times 100$$

The results were expressed also as Trolox equivalent antioxidant capacity (TEAC) calculated from a validated calibration curve of Trolox in NaCl 0.9% (5–35 µM), processed in the same way as the samples for comparative purposes.

The antioxidant capacity of a resveratrol solution in ethanol:water 10:90 *v*/*v* medium after being exposed to white light (HITACHI 8 W F8T5 daylight, Chiyoda, Japan) for 3 h (14 cm gap from the lamp) was also tested.

### 2.14. Statistical Analysis

The effects of hydrogel composition on swelling, drug loading, permeability through porcine eye tissues and biofilm formation were analyzed using ANOVA and multiple range test (Statgraphics Centurion XVII, StatPoint Technologies Inc., Warrenton VA, USA).

## 3. Results and Discussion

### 3.1. Hydrogel Synthesis

HEMA and silicone hydrogels were designed to combine the features demanded by CLs (water uptake, light transmittance, mechanical properties) while adding antibiofilm capacity, with the aim of attenuating the risk of ocular infections associated with CL wearing [15,19]. Two strategies were followed to endow CLs with antibiofilm features: copolymerization with MPC and loading of resveratrol. To the best of our knowledge, resveratrol-eluting CLs have not been previously investigated, although resveratrol may find applications in the ocular field as an antioxidant, anti-inflammatory, antiangiogenic and anticarcinogenic agent [33,46,47]. MPC is a component of one commercially available soft (HEMA-based) CL brand (Proclear^®^) that claims to retain more water on its surface. MPC is also being tested as a surface component of post-synthesis-modified CLs [14,15]. In the present study, MPC was added as comonomer during the synthesis of the hydrogels. The highest content in MPC was limited by the compatibility of this hydrophilic monomer with the silicone-based mixture. MPC can make the silicone hydrogels whitish due to the microphase separation of silicone and hydrophilic monomers [48], which was indeed evident for S5 and S6 hydrogels (as discussed below). Proclear^®^ 1 day CLs are reported to have 3% MPC [49], which is equivalent to 101 mM; therefore, this proportion was chosen as the highest one to be investigated. Hydrogel codes H1, H2, H3, H4, H5 and H6 in Table 1 correspond to 0, 10, 20, 30, 60 and 101 mM MPC; similarly, S1, S2, S3, S4, S5 and S6 correspond to 0, 10, 20, 30, 80 and 101 mM MPC.

### 3.2. Water Uptake and Wettability

The water uptake (Table 2 and Figure S3) was higher for silicone hydrogels than for HEMA hydrogels despite the hydrophobic character of TRIS. The addition of hydrophilic monomers, mainly NVP [50,51] and, to a lesser extent, MPC [17], explains the greater water uptake of silicone hydrogels. In fact, the designed silicone hydrogels showed a higher water content than that previously recorded for other silicone hydrogels [48,50], even with a very similar composition [52]. The water uptake of HEMA hydrogels was in good agreement with previously reported values [34,53] and was similar to that obtained for other HEMA hydrogels copolymerized with acrylic acid and 4-vinyl pyridine [54]. The amount of water absorbed for both types of hydrogels slightly increased with the addition of MPC. For a given composition, no significant changes in swelling were observed between water and SLF. Contrastingly, the percentage of swelling was statistically higher (*p* < 0.05) for hydrogels immersed in the resveratrol solution in ethanol:water 10:90 medium due to the presence of ethanol [55]. The highest value of water uptake was observed for Proclear^®^ 1 day CLs.

The hydrogels’ surface wettability was determined by measuring the contact angle using the captive bubble method [35]. Compared to the sessile drop method commonly used to characterize rigid gas permeable CLs, the captive bubble method is particularly advantageous for hydrogel materials that may lose water when exposed to air and may deform (swell) when the drop enters into contact with the surface. The captive bubble method is carried out with hydrogel pieces immersed in water (which prevents changes in swelling degree) and, although the recording of the bubble shape can be time-consuming, the measurements are more reliable [35]. The contact angle values were very similar for all compositions, in the range of 37° to 41° for HEMA hydrogels and 32° to 46° range for silicone ones (Figure S4). It is known that in the dry state, silicone-based hydrogels are more hydrophobic than HEMA-based ones, due to the presence of siloxane groups in the former [52]. However, upon hydration, the silicone-based hydrogel acquired a similar hydrophilicity to HEMA, since, in the presence of water (polar solvent), reorientation of the hydrophilic and hydrophobic groups of the chains may occur: the hydrophobic siloxane groups are mainly hidden, and the hydrophilic functionalities of HEMA and NVP become exposed to the surface. Although the addition of MPC led to higher water uptake values in both types of hydrogel, the measurements of water contact angle did not show a significant effect on surface hydrophilicity. This may be due to the small amount of MPC, whose effect is minor when compared to that induced by the other hydrophilic monomers present in the hydrogel matrix. Other authors found a reduction in the water contact angle with an increase in MPC amount, but for hydrogels with MPC grafted to the surface [56,57] or with much higher amounts of MPC [58]. It should be noted that the contact angles obtained for all compositions fell in the range of the values typical of commercial CLs and were similar to the contact angle reported for Proclear^®^ (47.4 ± 7.5°) [59] using the same technique.

### 3.3. Light Transmission

**Figure 2:**
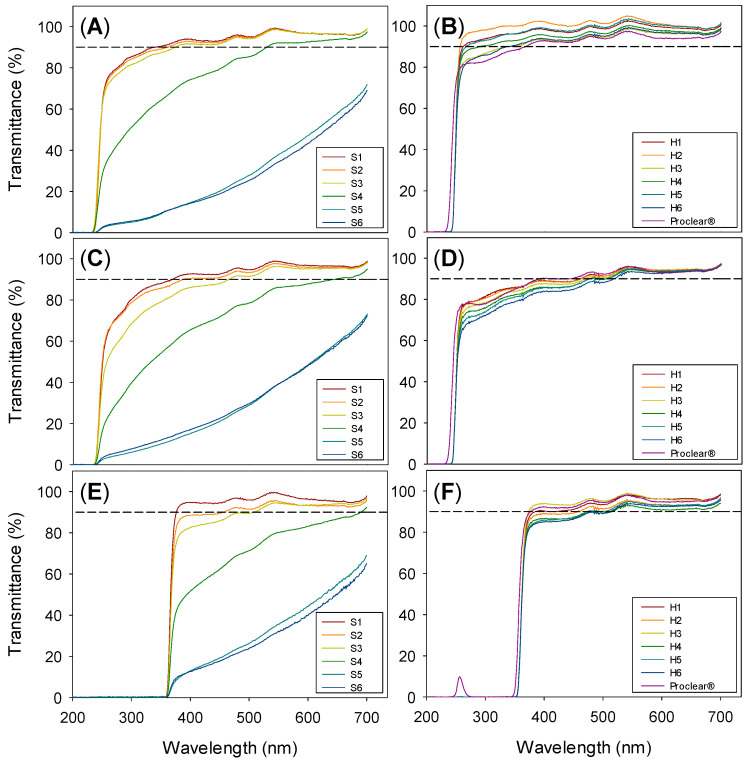
Light transmission of silicone (**left**) and HEMA (**right**) hydrogels swollen in water (**A**,**B**), SLF (**C**,**D**) and resveratrol loading solution (**E**,**F**). Dashed lines indicate the acceptance value of 90% transmittance.

Light transmittance of all hydrogel compositions in water, SLF and resveratrol solution (in ethanol:water 10:90 *v*/*v* medium) showed values above 90% in the visible range (600 nm), except for the S5 and S6 hydrogels, which were slightly opalescent (Figure 2; Figure S5). Although all hydrogels had an apparently smooth surface, SEM images of freeze-dried hydrogels recorded at high magnification evidenced the roughness of S5 and S6 hydrogels, showing islet-like patterns typical of microphase separation (Figure S6) [15]. S4 images were quite similar to those of S1, which suggests that the low MPC content did not trigger phase separation. No significant differences in light transmittance were observed between hydrogels swollen in water, SLF or resveratrol solution, besides the fact that the loading of resveratrol provided very efficient protection against UV radiation (Figure 2E,F). The beneficious UV filter effect had a nondetrimental impact on the light transmission above 400 nm.

### 3.4. Mechanical Properties

The Young’s modulus values of the swollen strips are shown in Table 2 for all hydrogel compositions. Silicone hydrogels had a Young’s modulus larger than HEMA hydrogels, in agreement with the behavior reported in the literature [60]. The Young’s modulus of the HEMA hydrogels was in the range of those typical of soft contact lenses [58]. The Young’s modulus registered for Proclear^®^ 1 day CLs matched the value of the data sheet (0.4 MPa). The values recorded for the silicone hydrogels were close to the first-generation silicone hydrogels, although it should be noted that, at the eye temperature, the values may be slightly lower [60]. Interestingly, MPC at the highest proportion investigated only caused a minor decrease (not statistically significant) in the mechanical properties of the CLs, in contrast to the previously reported decrease in Young’s moduli observed for HEMA–MPC networks prepared with larger proportions of MPC [58].

### 3.5. Resveratrol Loading and Release

**Figure 3:**
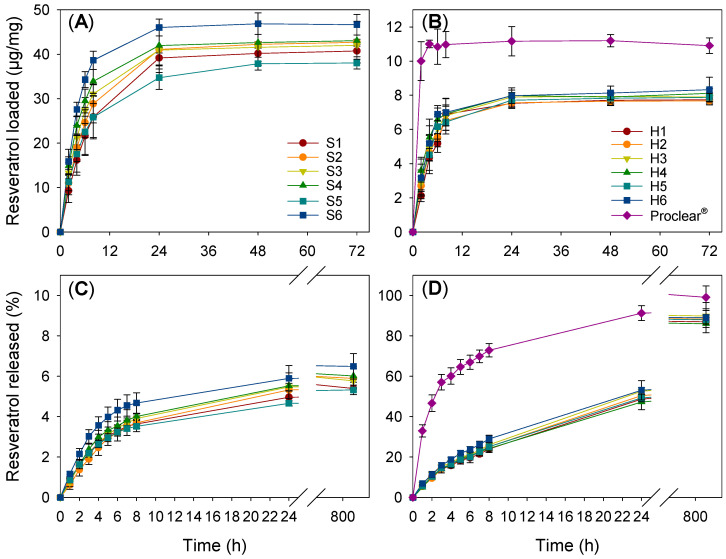
Resveratrol loading profiles for silicone (**A**) and HEMA (**B**) based hydrogels at 36 °C and 180 rpm for 72 h, and release profiles from silicone (**C**) and HEMA (**D**)-based hydrogels in NaCl 0.9% medium at 36 °C.

Resveratrol loading was carried out by soaking the hydrogel discs in a resveratrol solution (100 µg/mL) in ethanol:water 10:90 *v*/*v* medium until no changes in the absorbance of the loading medium were observed. Loading of the hydrogels during polymerization by the addition of resveratrol to the monomer solution was discarded since the antioxidant power of resveratrol may have hindered the polymerization [37]. All hydrogels were soaked for at least 3 days, but equilibrium was attained in less than 48 h (Figure 3A,B). Silicone hydrogels captured more than 75% resveratrol initially present in the loading solution (estimated from the difference between resveratrol amount in the loading medium at time 0 and at the end of the test). HEMA hydrogels rapidly sorbed resveratrol, and the final loading corresponded to ~44% resveratrol available. To monitor the loading, aliquots of the medium were taken for subsequent dilution before absorbance measurements. This caused some loss of resveratrol available for loading. Thus, a second loading study was carried out without intermediate measurements for 72 h; the amount loaded by silicone hydrogels corresponded to more than 80% resveratrol initially present in the loading solution and to ~50% for HEMA hydrogels (data shown in Table 2). Compared to the designed HEMA hydrogels, Proclear^®^ 1 day CLs showed higher loading, as expressed per unit of weight. The greater surface contact area with the Proclear^®^ 1 day CLs (14.2 mm diameter), and the lower thickness (center thickness of 0.09 mm) compared to the HEMA hydrogels (10 mm diameter, 0.45 mm thickness), may have contributed to faster and more efficient loading. Nevertheless, the total amount loaded per CL was lower for Proclear^®^ 1 day CLs than for the other HEMA hydrogel discs due to the lower weight of the CLs.

Overall, both types of hydrogel had high affinity for resveratrol, but the network/water partition coefficient for silicone hydrogels (K_N/W_ in the 373 to 492 range) was four/six-fold higher than that recorded for HEMA hydrogels (K_N/W_ in the 77–81 range). Proclear^®^ 1 day CLs had K_N/W_ values (~100) slightly higher than those recorded for HEMA hydrogels. These values are in the range of or even greater than those previously reported for related silicone hydrogels [61] and HEMA hydrogels [62] in the presence of other active ingredients. In any case, the large K_N/W_ values obtained indicated that resveratrol was loaded not only in the aqueous phase of the hydrogel but also interacting with the network [37]. No evident effect of MPC addition on resveratrol loading was found.

Resveratrol release profiles from both types of hydrogel were remarkably different (Figure 3C,D). The test was carried out in Falcon^®^ tubes with a sufficient volume of liquid in order to avoid saturation (resveratrol solubility in NaCl 0.9% was quantified as 27.4 (s.d. 1.4) µg/mL) and under gentle stirring (180 rpm) in order to avoid pseudo-equilibrium and artifact plateaus. Reliable in vitro release methods for drug-loaded CLs that can serve to predict drug release in vivo are still needed [63]. Nevertheless, a requirement that any in vitro method must meet is to avoid the occurrence of false balances between the drug remaining in the contact lens and the drug already delivered, leading to delivery rates much slower than would be expected in vivo.

Silicone hydrogels strongly retained resveratrol and released less than 8% of the amount loaded in five weeks. Contrastingly, HEMA hydrogels provided sustained release of 33% load in the first 8 h. The amount of resveratrol released from Proclear^®^ was above 80% in the first 8 h. In comparison with other similar silicone-based hydrogels that were loaded with chlorhexidine, moxifloxacin and diclofenac [64], the release was slower, and no burst was recorded. This finding suggests more intense hydrophobic interactions between the silicone network and resveratrol.

The amount of resveratrol released was above the minimum required (2.28 µg/mL) in cell cultures to protect retinal pigment epithelial cells from UVA-induced oxidative damage [65] and also to protect retinal pigment epithelial cells against hyperglycemia-induced inflammation and gap junction intercellular communication degradation [66]. Thus far, no resveratrol-loaded CLs have been described in the literature for the ophthalmic administration of resveratrol, and most information relies on its topical ocular [67] and oral administration through food or dietary supplements [68,69,70]. Although the minimum effective concentration of resveratrol needed in the tear fluid for therapeutic effects is unknown, assuming that the concentration reported above (2.28 µg/mL) is sufficient and considering that the volume of tear produced per day is 4.32 mL (i.e., ~3 µL/min), the minimum amount that the CL should supply is 9.84 µg. Assuming that the weight of a common CL is 13 mg, all designed hydrogels can easily provide more resveratrol than the minimum required (9.84/13 = 0.75 µg/mg) after the first 2 h of wearing, according to the release profiles shown in Figure 3.

Regarding the minor effect of MPC on the resveratrol loading and release results, only formulations prepared without (S1, H1) and with the highest proportions of MPC (S4, S5, S6 and H4, H5 and H6) were considered for subsequent studies.

### 3.6. Resveratrol Stability

**Figure 4:**
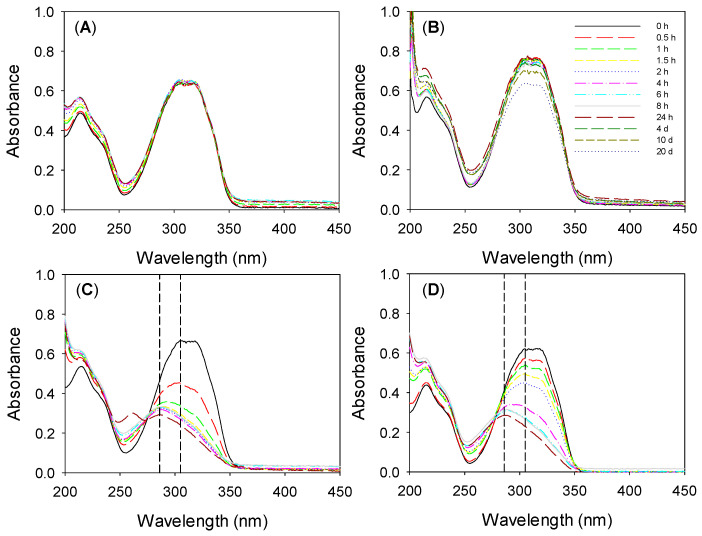
UV–Vis spectra recorded for resveratrol in ethanol:water 10:90 *v*/*v* medium (**A**) and resveratrol in NaCl 0.9% (**B**) without light at 36 °C and 180 rpm for several days, and resveratrol in ethanol:water 10:90 *v*/*v* medium exposed to white light (**C**) and working area light (**D**) for 24 h. Dashed lines indicate the maximum wavelength of *cis*-resveratrol (286 nm) and *trans*-resveratrol (305 nm).

Since *trans*-resveratrol is quite prone to isomerization and degradation [36], stability studies were carried out to gain an insight into the feasibility of using resveratrol for topical ophthalmic administration. No changes in the UV–Vis spectra of resveratrol in ethanol:water 10:90 *v*/*v* medium and in NaCl 0.9% solution were recorded for 24 h and 10 days, respectively, under the loading and release conditions (dark, 36 °C and 180 rpm) (Figure 4A,B). When resveratrol loading solution was exposed to white light for 24 h (Figure 4C,D), the absorbance at 305 nm (maximum for *trans*-resveratrol) decreased, and thus the ratio of absorbance at 286 nm (maximum of *cis*-resveratrol) to absorbance at 305 nm increased, which indicated its transformation into the less active isomer [71]. This phenomenon occurred more slowly when the resveratrol solution was exposed to the usual light conditions of the laboratory (Figure 4D).

**Figure 5:**
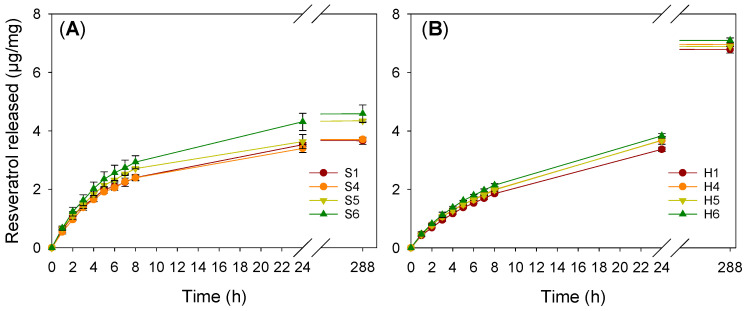
Release profiles of resveratrol in NaCl 0.9% from silicone- (**A**) and HEMA (**B**)-based hydrogels. After being loaded with resveratrol, the hydrogels were exposed for 3 h to white light. The release profiles were constructed considering the absorbance maximum of *trans*-resveratrol.

To investigate whether the hydrogels could protect resveratrol from light degradation, resveratrol-loaded HEMA- and silicone-based hydrogels (by soaking in ethanol:water solution for 72 h as in Section 3.5) were removed from the loading medium and directly placed into empty quartz cells and exposed to a white light lamp (HITACHI 8 W F8T5 daylight, Japan) for 3 h at room temperature. Then, the release profiles were recorded (Figure 5); the UV–Vis patterns were very similar, as well as the amounts released, to those recorded for hydrogels that were not exposed to the white light (Figure 3). This suggested that the hydrogels could protect resveratrol against photodegradation, as later confirmed by the antioxidant tests (Section 3.10).

### 3.7. Corneal and Scleral Permeability

The permeability and retention capacity of resveratrol released from the most promising hydrogels and Proclear^®^ 1 day CLs were investigated using porcine eyes, which are the most similar to human eyes considering the globe size, corneal thickness, ratio of globe diameter to corneal length, presence of Bowman’s layer, sclera histology and collagen bundle organization [72]. As a control, a resveratrol concentrated solution (70 µg/mL in ethanol:water 10:90 *v*/*v* medium) was used.

**Figure 6:**
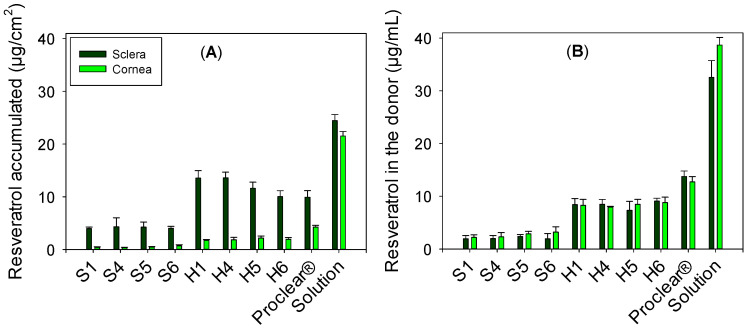
Amounts of resveratrol accumulated in cornea and sclera (**A**) and remaining in the donor chamber (**B**) when delivered as resveratrol-loaded silicone and HEMA hydrogels, and Proclear^®^ contact lenses, or as resveratrol solution (70 µg/mL).

Resveratrol crossed neither the cornea nor the sclera, regardless of whether it was released from the hydrogels or directly applied as a concentrated solution. Resveratrol solubility in NaCl 0.9% was measured to be 27.4 (s.d. 1.6) µg/mL and the experimental setup ensured that the receptor chamber was not saturated. Therefore, a sufficient concentration gradient could exist between the donor and the receptor chamber. For example, when the concentrated resveratrol solution was tested, the donor chamber contained 1 mL of 70 µg/mL (in ethanol:water 10:90 *v*/*v*). Since the volume of receptor medium was 6 mL, if all resveratrol could pass to the receptor (assuming no adsorption to the cornea or sclera), the maximum concentration that could be reached in the receptor would be 10 µg/mL. Therefore, the absence of resveratrol in the receptor chamber cannot be attributed to low solubility in the receptor medium. In the 6 h time frame of the study, measurable amounts of resveratrol in the receptor chamber were not recorded in any case. Contrastingly, resveratrol accumulated in the cornea and sclera tissue (Figure 6A). Accumulation was higher in the sclera than in the cornea for all formulations. The highest amount accumulated was recorded for the resveratrol solution (24.5 ± 1.2 and 21.6 ± 0.8 µg/cm^2^ for sclera and cornea, respectively). This finding clearly correlated with the lower resveratrol levels that the hydrogel formulations can provide to the donor chamber, as shown in Figure 6B. In good agreement with the release profiles shown in Figure 3, HEMA hydrogels provided more resveratrol to the donor chamber than silicone hydrogels.

Differences in silicone hydrogel composition did not cause any change in the amount of resveratrol accumulated in the cornea or sclera (no statistically significant differences for a given tissue). For H1 and H4 hydrogels, the amount of resveratrol accumulated in the sclera (13.6 ± 1.4 and 13.6 ± 1.1 µg/cm^2^) was statistically higher than for H6 and Proclear^®^ (10.1 ± 1.1 and 9.9 ± 1.2 µg/cm^2^). Regarding cornea tests, the amount of resveratrol accumulated was, surprisingly, higher for Proclear^®^ CLs, which may be related to the faster release provided by these CLs. Interestingly, the amounts of resveratrol accumulated in the cornea and sclera once released from HEMA hydrogels were remarkably higher than those previously reported for the antioxidant transferulic acid [37].

### 3.8. HET-CAM Test

The Hen’s Egg Test on the Chorioallantoic Membrane (HET-CAM) assay was used to gain an insight into the compatibility of the developed hydrogels with the ocular surface. The vasculature of the CAM of fertilized eggs is comparable to the conjunctiva structure [39]. All hydrogel compositions could be considered non-irritating because none of them caused hemorrhage, vascular lysis or coagulation of CAM vessels during the 5 min of the test (Figure S7) [62]. The IS registered for the positive control was 19.4.

### 3.9. Protein Adsorption

The adsorption of albumin and lysozyme, as two of the major proteins present in the tear fluid, was evaluated using a QCM-D. Changes in the values of frequency (∆f/n) and dissipation (∆D) are shown in Figures S8–S11 (Supporting Information) and summarized in Table 3 for the third harmonic.

The addition of lysozyme and albumin led to a decrease in frequency in all cases, demonstrating that both proteins adsorbed onto the surfaces. The decrease in frequency was more accentuated in the case of lysozyme, independently of the nature of the hydrogel (silicon-based or HEMA-based), indicating that a higher amount of protein adsorbed onto the hydrogel’s surface. In all cases, the adsorption was attenuated with the increase in the MPC amount in the hydrogels, which confirmed the capacity of MPC to avoid protein adsorption [73]. This effect was more pronounced for lysozyme on HEMA-based hydrogels.

The degree of overlapping of the curves Δf/n versus time obtained for the different harmonics was analyzed to predict the viscoelastic character of the adsorbed film. The obtained graphics presented a high degree of overlapping, indicating that the protein adsorbed layers showed high stiffness. The rigid nature of the formed films was confirmed by the low values of ΔD, which indicates that a low amount of energy was dissipated.

For rigid films, the variation in frequency (Δf/n) is proportional to the mass of the adsorbed film (Δm) according to the Sauerbrey Equation (3):

(3) $$\Delta m=-C\times \frac{\Delta f}{n}$$

where C is the sensitivity constant based on the physical properties of the quartz crystal (C = 17.7 ng cm^−2^ Hz^−1^ for a 5 MHz crystal) [74,75]. Additionally, the thickness (d_eff_) of the adhering films can be estimated through Equation (4), considering 1.15 g/cm^3^ [75] and 1.38 g/cm^3^ [76] as the density of albumin and lysozyme films [77]:

(4) $$d_{\mathrm{eff}}=\frac{\Delta m}{\rho_{\mathrm{eff}}}$$

Overall, MPC demonstrated a significant antifouling effect for both proteins: for the hydrogels with the highest value of MPC, lower thickness and Δm values were obtained. This is in agreement with previous studies that found that the existence of zwitterionic MPC moieties on the surfaces of the HEMA- and silicone-based hydrogels decreased the adsorption of proteins, such as lysozyme, fibrinogen and albumin [56,57,58]. This protein repulsion capacity was attributed to the high hydration of the phosphorylcholine groups present on MPC [56,58]. It is known that the increase in the ratio of MPC increases the amount of free water but decreases that of bound water [78]. Therefore, when a protein comes into contact with the hydrogel surface, it may remain in this native state, not altering its structure. This facilitates the release of loosely bound protein molecules, leading to a decrease in protein adsorption.

The differences between the adsorbed amounts of albumin and lysozyme may be related to the size, shape, charge, conformational stability of the proteins and their concentration in the adsorbing solution. Protein adsorption is a complex phenomenon, and the weight of the different contributions is difficult to identify. Albumin is a large, anionic, soft protein (MW 66.4 kDa) with a heart shape (8 mm side × 3 nm thickness) while lysozyme is a small, ellipsoid, cationic, hard protein (MW 14.7 kDa) with axes of 2.6 nm and 4.5 nm [79]. In this work, different concentrations of each protein were used to perform the adsorption experiments (0.05 mg/mL for albumin and 1.9 mg/mL for lysozyme). Both conformational and concentration differences may explain the lower amount of adsorbed albumin on the hydrogels.

The inhibitory effect of MPC on protein adsorption was less evident for lysozyme on silicon-based hydrogels, which may be due to the stronger interactions between the protein and the surface. In fact, the hydrogel surface should be negatively charged (high affinity to positive polyelectrolytes was previously found [80]), enhancing the binding to the positively charged protein. Such a preferential binding of lysozyme on MPC-containing hydrogels may readily occur after placement on the eye’s surface [81], which may contribute to the antimicrobial performance [82].

### 3.10. Antibiofilm Properties

**Figure 7:**
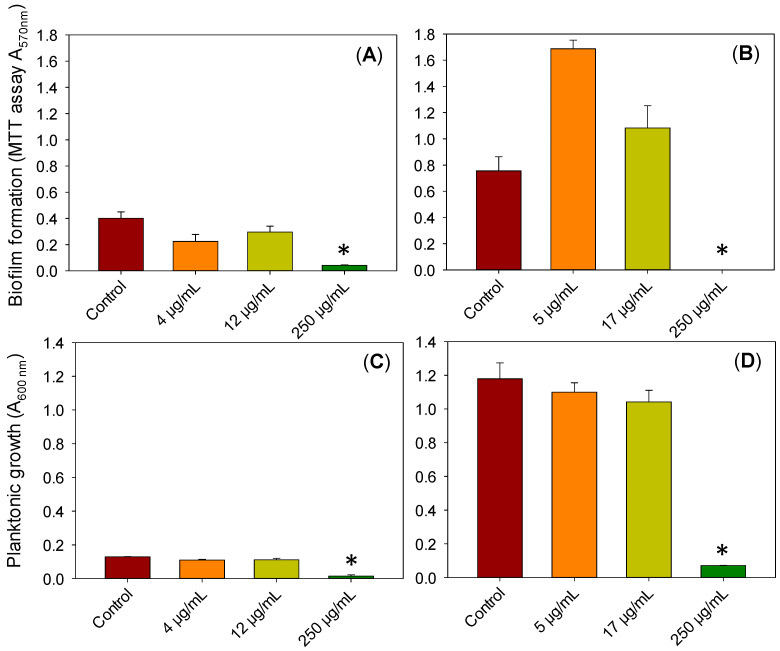
Growth of *Pseudomonas aeruginosa* (**A**,**C**) and *Staphylococcus aureus* (**B**,**D**) biofilm on the surface of glass coverslips measured with the MTT assay (**A**,**B**) and in the culture medium surrounding the coverslips (**C**,**D**), to which resveratrol at different concentrations was added. * indicates a statistically significant difference with respect to the control without resveratrol (*p* < 0.05).

First, the susceptibility to soluble resveratrol of the two main causal agents of ocular infections, *S. aureus* and *P. aeruginosa* [5], was evaluated by quantifying the effect of different concentrations of resveratrol on bacteria growth and biofilm formation on inert glass surfaces. *P. aeruginosa* biofilm formation on the surface of the coverslips immersed in growth medium supplemented with resveratrol was lower than without resveratrol (Figure 7A), although the differences were not statistically significant for the two lower concentrations tested (4 and 12 µg/mL). The biofilm formation significantly decreased for a 250 µg/mL resveratrol solution, showing the capacity of resveratrol to inhibit biofilm formation against *P. aeruginosa* and also to prevent the growth of planktonic bacteria (Figure 7C). These findings agree well with previous reports on Resveramax™ (oily alimentary supplement) that evidenced that resveratrol was active against *P. aeruginosa* PAO1 biofilm [26,83]. In contrast, the biofilm formation after 48 h of growth of *S. aureus* (Figure 7B) was higher for 5 µg/mL of resveratrol than for the controls, but the biofilm was completely inhibited when the resveratrol concentration was raised to 250 µg/mL. The bacterial growth in the medium surrounding the coverslips was in good agreement with the absorbances recorded for the surfaces (Figure 7D). The increase in biofilm formation observed at low resveratrol concentrations for *S. aureus* may be the result of a stress response of the cells in the presence of sub-inhibitory concentrations of this compound, since the slower growth of bacteria in the biofilm may have had a protective effect against resveratrol. An increase in biofilm formation as a response to the cell stress caused by antimicrobial agents has been described before [84].

In all cases, it should be noted that the MTT assay used to evaluate the biofilm formation determines mitochondrial activity since it is based on the conversion of MTT into formazan crystals by living cells and not only biofilm biomass or cell number, being therefore a highly sensitive method [85]. Resveratrol has been suggested to alter the MTT reduction rate in mammalian cell cultures, not because of a direct effect on MTT reduction but through an indirect effect on cell metabolism [86]. Such an effect is of small magnitude and has not been reported for bacteria.

**Figure 8:**
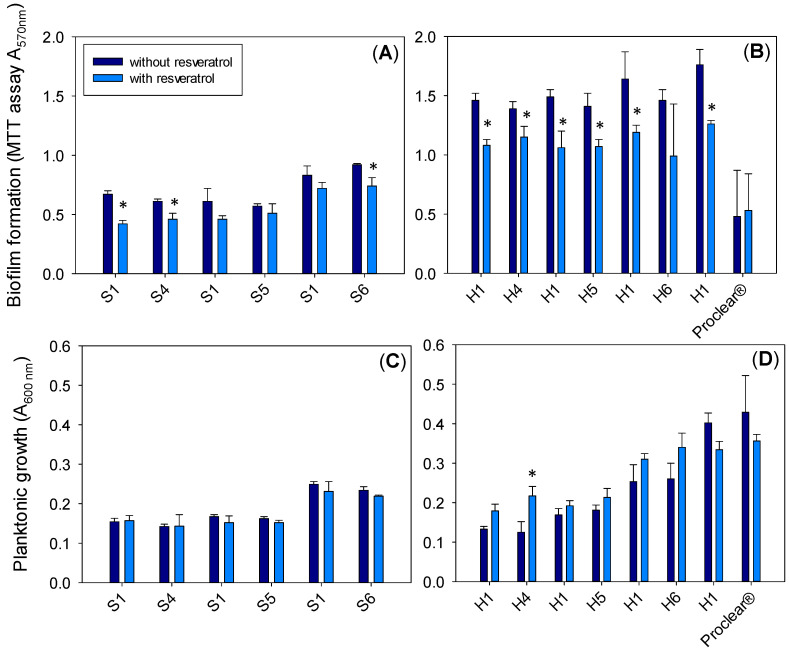
Growth of *Pseudomonas aeruginosa* PAO1 biofilm on the surfaces of silicone (**A**) and HEMA (**B**) hydrogels (without and with resveratrol) measured with the MTT assay, and in the culture medium surrounding the silicone (**C**) and HEMA (**D**) hydrogels (without and with resveratrol) after 6 h of incubation. * indicates a statistically significant difference between hydrogels with and without resveratrol (*p* < 0.05). In each experiment, a silicone hydrogel prepared with MPC (S4, S5 and S6) was evaluated in parallel to the silicone hydrogel without MPC (S1). Similarly, the HEMA hydrogels prepared with MPC (H4, H5 and H6) were evaluated in parallel to the HEMA hydrogel without MPC (H1).

Next, the antibiofilm performance of silicone and HEMA hydrogels with and without MPC in their composition and loaded or not with resveratrol was investigated. In the case of *P. aeruginosa*, more biofilm was formed on the hydrogels without resveratrol (Figure 8A,B) compared to those loaded with resveratrol. No differences were recorded for Proclear^®^ 1 day CLs with and without resveratrol, which may be related to the fact that the amount loaded per lens was the lowest (see Table 2). In HEMA hydrogels, the inhibitory effect of the resveratrol loading was more evident, probably due to the greater amounts released. The absorbance of the supernatants was also measured (Figure 8C,D) and the planktonic bacterial growth was very similar in the culture media around the hydrogels loaded or not with resveratrol. Notably, although the biofilm formation was clearly lower in the resveratrol-loaded HEMA hydrogels than in their respective unloaded controls (Figure 8B), planktonic growth was generally higher in the presence of resveratrol-loaded lenses (Figure 8D), indicating the specific antibiofilm activity of the compound for *P. aeruginosa*.

**Figure 9:**
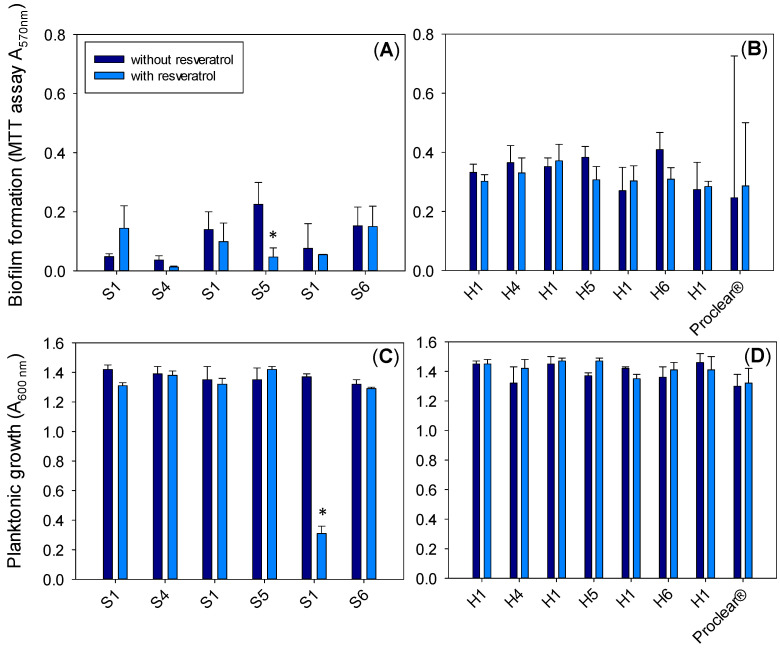
Growth of *Staphylococcus aureus* biofilm on the surface of silicone (**A**) and HEMA (**B**) hydrogels (without and with resveratrol) and in the medium surrounding the silicone (**C**) and HEMA (**D**) hydrogels (without and with resveratrol) after 48 h of incubation. In each experiment, a silicone hydrogel prepared with MPC (S4, S5 and S6) was evaluated in parallel to the silicone hydrogel without MPC (S1). Similarly, the HEMA hydrogels prepared with MPC (H4, H5 and H6) were evaluated in parallel to the HEMA hydrogel without MPC (H1). * indicates a statistically significant difference between hydrogels with and without resveratrol (*p* < 0.05).

In the case of *S. aureus*, the biofilm formation after 48 h of incubation (Figure 9A,B) for all hydrogels was lower than for *P. aeruginosa* despite the much higher bacterial growth in the medium surrounding the hydrogels (Figure 9C,D). For all HEMA- and silicone-based hydrogels, the biofilm formation was almost the same and no statistically significant differences were observed with the addition of MPC and/or resveratrol. This finding agreed with two other studies where resveratrol did not reduce biofilm formation and confirms that strain variation and assay conditions may influence the efficacy [87,88]. Unexpectedly, no remarkable antibiofilm effect could be assigned to MPC, which could be related to the fact that, in the designed hydrogels, the MPC monomer was copolymerized during the synthesis process and not added later as a surface modification process, as commonly reported in the literature [89]. Therefore, MPC is expected to be evenly distributed in the bulk of the hydrogel and not confined to the surface. This may have caused the density of MPC chains on the surface to be insufficient to prevent bacteria adhesion.

The adhesion of bacteria is usually higher on hydrophobic hydrogels than on hydrophilic ones [3,90,91,92]. Here, the biofilm formation for both bacteria was lower for silicone hydrogels. Although the wettability of the silicone- and HEMA-based hydrogels was not significantly different, the lower biofilm formation could be related to the higher water uptake observed for silicone hydrogels due to the presence of hydrophilic monomers (mainly NVP), as explained above.

### 3.11. Antioxidant Properties

The DPPH assay was used to check whether resveratrol maintained its antioxidant activity after being loaded and released from HEMA and silicone hydrogels. Freshly prepared NaCl 0.9% release medium, as well as NaCl 0.9% solutions in which non-loaded hydrogels were soaked, were also tested to verify that there were no leaching substances that could cause false antioxidant activity during the assay. The DPPH• scavenging capacity was expressed as µg/mL in the reaction medium, as a percentage, as explained previously [44], and as Trolox equivalent antioxidant capacity (TEAC) for comparative purposes. Analysis of freshly prepared resveratrol loading solution led to 3.5 ± 1.2 µg/mL DPPH levels and 40.5 ± 2.8 µM TEAC. Samples from the release tests are expected to cover a wide range of resveratrol concentrations (according to Figure 3 and Figure 5) but well below the concentration of the loading solution.

According to the results (Table 4, Figures S12–S14), the levels of DPPH• radicals registered for NaCl 0.9% medium without resveratrol (20.1 µg/mL) were higher than for the release medium of the resveratrol-loaded hydrogels. This finding indicates that the released resveratrol maintained its capacity to reduce the free radicals and thus retained its antioxidant activity after being loaded and released from the HEMA and silicone hydrogels. At the same time, the effect registered was very similar within the same type of hydrogel, but antioxidant capability was higher for HEMA than for silicone hydrogels, as expected, since HEMA hydrogels released higher amounts of resveratrol into the medium. Non-loaded hydrogels showed values of TEAC that were close to zero or even negative, meaning that they did not release any substance that may have interfered with the test.

The antioxidant capacity of resveratrol solutions after being exposed to white light (HITACHI 8 W F8T5 daylight, Japan) for 3 h was confirmed despite isomerization from *trans* to *cis* (13.2 ± 0.03 µg/mL DPPH levels, and 16.37 ± 0.07 µM TEAC). Overall, both HEMA and silicone hydrogels helped resveratrol to maintain its antioxidant activity, which may be useful in managing ocular diseases that benefit from a decrease in reactive oxygen species (ROS) levels.

## 4. Conclusions

The use of MPC as a comonomer of HEMA- and silicone-based hydrogels and their loading with resveratrol have been studied here for first time to obtain hydrogels with antioxidant and antibiofilm properties. MPC increased the water uptake and decreased the amount of protein adsorbed while preserving the mechanical properties of the hydrogels. Only silicone hydrogels prepared with the highest proportions of MPC investigated (80 and 101 mM) evidenced a decrease in light transmission. All hydrogels were able to uptake relevant amounts of resveratrol. The higher affinity of silicone hydrogels for resveratrol, probably due to hydrophobic interactions, notably increased the amount loaded but also caused the release to occur more slowly. As a consequence, the amounts of resveratrol accumulated in the cornea and sclera were lower when delivered from the silicone hydrogels. Regarding the antibiofilm activity, resveratrol decreased biofilm formation by *P. aeruginosa*, but no protective effect was recorded for MPC against the two strains investigated. Nevertheless, the fact that the hydrogels prepared with MPC showed preferential sorption of lysozyme with respect to albumin may contribute to the antibacterial effects in vivo, which should be evaluated in future studies. The inconclusive antibiofilm performance of the hydrogels on *S. aureus* suggests that the loading should be increased in order to achieve an inhibitory concentration. Importantly, HEMA- and silicone-based hydrogels preserved the antioxidant activity of resveratrol and showed a protective effect against photodegradation. Overall, hydrogels containing MPC and loaded with resveratrol are demonstrated to be suitable candidates for the preparation of CLs with antibiofilm and antioxidant performance.

## Figures and Tables

**Table 1:** Hydrogels composition *.

Hydrogel	Code	MPC (mg)	TRIS (mL)	NVP (mL)	HEMA (mL)	EGDMA (µL)	AIBN (mg)
Silicone	S1	0	1.58	2.50	0.92	32	12.50
	S2	14.76	1.58	2.50	0.92	32	12.50
	S3	29.52	1.58	2.50	0.92	32	12.50
	S4	44.29	1.58	2.50	0.92	32	12.50
	S5	118.1	1.58	2.50	0.92	32	12.50
	S6	150.0	1.58	2.50	0.92	32	12.50
HEMA	H1	0	-	-	4	6.04	6.57
	H2	11.81	-	-	4	6.04	6.57
	H3	23.62	-	-	4	6.04	6.57
	H4	35.43	-	-	4	6.04	6.57
	H5	70.90	-	-	4	6.04	6.57
	H6	120.0	-	-	4	6.04	6.57

* MPC: 2-methacryloyloxyethyl-phosphorylcholine, TRIS: 3-(methacryloyloxy)propyltris (trimethylsiloxy)silane; NVP: N-vinylpyrrolidone; HEMA: 2-hydroxyethyl methacrylate; EGDMA: ethyleneglycol dimethacrylate; AIBN: 2,2′-azobis(2-methylpropionitrile).

**Table 2:** Water uptake at equilibrium and Young’s modulus (mean values ± S.D.) for all non-loaded silicone hydrogels (S1–S6) and HEMA hydrogels (H1-H6) immersed in water at room temperature and amounts of resveratrol loaded when the hydrogels were soaked in a resveratrol solution (100 µg/mL) in ethanol:water 10:90 *v*/*v* medium at 36 °C and 180 rpm for 72 h without intermediate measurements.

Hydrogel Code	Water Uptake (%)	Young’s Modulus (MPa)	Resveratrol Loaded (µg/mg)
S1	91.9 ± 0.6	2.00 ± 0.07	37.37 ± 0.68
S2	92.2 ± 0.5	2.29 ± 0.17	49.22 ± 1.74
S3	93.2 ± 1.1	2.01 ± 0.37	41.66 ± 0.96
S4	92.4 ± 1.5	2.46 ± 0.09	47.87 ± 0.72
S5	105.8 ± 0.9	2.47 ± 0.09	50.11 ± 2.06
S6	107.3 ± 0.9	2.32 ± 0.21	58.58 ± 4.05
H1	56.4 ± 0.5	0.61 ± 0.03	8.57 ± 0.56
H2	57.4 ± 0.3	0.67 ± 0.01	8.80 ± 0.35
H3	58.4 ± 0.6	0.68 ± 0.04	8.32 ± 0.22
H4	59.2 ± 0.1	0.67 ± 0.05	8.66 ± 0.53
H5	62.6 ± 0.8	0.66 ± 0.01	9.27 ± 0.75
H6	67.6 ± 0.1	0.60 ± 0.01	8.40 ± 0.09
Proclear^®^ 1 day	139.9 ± 2.3	0.44 ± 0.08	13.59 ± 0.36

**Table 3:** Frequency (∆f/n) and dissipation (∆D) variations for the 3rd harmonic for albumin and lysozyme adsorption onto silicone and HEMA hydrogels, obtained by QCM-D. Estimated values for mass variation (∆m) and layer thickness (d_eff_) are also presented (average ± standard deviation, *n =* 4).

Hydrogel	Protein	∆f/n (Hz)	∆D (×10^−6^)	∆m (ng/cm^2^)	d_eff_ (nm)
S1	Albumin	−15.5 ± 4.1	0.9 ± 0.6	275 ± 72	2.4 ± 0.6
S4		−7.0 ± 1.5	0.9 ± 1.1	124 ± 27	1.1 ± 0.2
S5		−4.3 ± 0.5	−0.2 ± 0.6	77 ± 9	0.6 ± 0.1
S6		−3.8 ± 0.8	−0.9 ± 0.4	68 ± 15	0.5 ± 0.1
S1	Lysozyme	−31.8 ± 6.3	0.4 ± 0.6	562 ± 110	4.1 ± 0.8
S4		−31.0 ± 3.4	0.6 ± 0.2	548 ± 60	4.0 ± 0.4
S5		−24.1 ± 7.8	0.1 ± 0.1	427 ± 140	3.1 ± 1.0
S6		−21.3 ± 0.6	0.9 ± 0.5	377 ± 11	2.7 ± 0.1
H1	Albumin	−15.6 ± 6.4	0.6 ± 0.3	275 ± 114	2.4 ± 1.0
H4		−14.1 ± 5.4	0.1 ± 1.1	250 ± 96	2.2 ± 0.8
H5		−8.5 ± 1.7	0.9 ± 0.1	150 ± 30	1.5 ± 0.4
H6		−6.4 ± 0.8	0.1 ± 0.9	114 ± 15	1.0 ± 0.1
H1	Lysozyme	−29.0 ± 4.7	1 ± 0.3	513 ± 83	3.7 ± 0.6
H4		−24.0 ± 7.3	0.1 ± 0.5	424 ± 129	3.1 ± 0.9
H5		−19.5 ± 2.2	1.4 ± 0.6	345 ± 39	2.5 ± 0.3
H6		−8.5 ± 2.4	1.8 ± 1.5	151 ± 43	1.1 ± 0.3

**Table 4:** DPPH• levels (µg/mL), DPPH scavenging effect (%) and Trolox equivalent antioxidant capacity (TEAC). All data are mean ± SDs (*n =* 3).

Hydrogels	DPPH• (µg/mL)		DPPH• Scavenging Effect (%)		TEAC (µM)	
	12 h	24 h	12 h	24 h	12 h	24 h
S1	15.6 ± 0.2	14. 8 ± 0.9	26.1 ± 1.1	29.0 ± 4.3	10.6 ± 0.5	12.6 ± 2.2
S2	16.3 ± 2.3	13.4 ± 0.6	27.8 ± 2.4	41.8 ± 2.6	8.9 ± 5.6	15. 9 ± 1.4
S3	15.6 ± 0.6	14.2 ± 0.3	38.7 ± 2.6	27.6 ± 1.5	10.5 ± 1.6	14.0 ± 0.7
S4	15.7 ± 2.1	13.2 ± 0.3	30.3 ± 9.6	33.4 ± 1.7	10.3 ± 5.2	16.5 ± 0.8
S5	15.1 ± 1.3	13.2 ± 0.7	35.5 ± 5.7	45.3 ± 3.1	11.8 ± 3.2	16.5 ± 1.8
S6	14.6 ± 0.9	13.6 ± 1.7	26.0 ± 4.9	36.8 ± 7.9	13.1 ± 2.3	15.6 ± 4.1
H1	11.5 ± 0.1	10.0 ± 1.0	44.0 ± 0.7	51.6 ± 4.9	20.5 ± 0.3	24.4 ± 2.4
H2	12.1 ± 0.8	11.9 ± 2.1	39.1 ± 4.3	40.2 ± 11.0	19.2 ± 2.0	19.6 ± 5.3
H3	13.0 ± 0.9	11.6 ± 1.7	36.4 ± 4.4	42.0 ± 8.8	17.0 ± 2.2	20.5 ± 4.2
H4	12.2 ± 0.4	10.8 ± 0.7	41.7 ± 2.0	47.7 ± 3.3	19.1 ± 1.0	22.3 ± 1.6
H5	11.3 ± 0.5	12.1 ± 3.6	48.1 ± 2.4	38.4 ± 18.9	21.3 ± 1.2	19.2 ± 8.9
H6	11.3 ± 0.8	9.8 ± 0.5	44.0 ± 4.1	58.9 ± 2.1	21.1 ± 2.0	24.9 ± 1.2
Proclear^®^	11.0 ± 0.4	11.1 ± 1.4	47.5 ± 2.0	46.5 ± 7.0	22.0 ± 1.0	21.6 ± 3.5

## Data Availability

Raw data is available upon request.

## Supplementary Materials

The following are available online at https://www.mdpi.com/article/10.3390/pharmaceutics13040532/s1. Figure S1: Image of a bubble adhered to a hydrogel contact lens when the captive bubble method was used for the measurement of the water contact angle (θ); Figure S2: Amsterdam Active Attachment model assembled with the tested hydrogel pieces; Figure S3: Water uptake (%) of silicone and HEMA hydrogels in water, SLF and resveratrol loading solution; Figure S4: Water contact angles on the silicone-based hydrogels and HEMA-based hydrogels; Figure S5: Reading test for silicone and HEMA hydrogels; Figure S6: SEM images of freeze-dried hydrogels recorded at high magnification (50,000×); Figure S7: Images of the HET-CAM test; Figures S8–S11: Normalized shift in the frequency, Δf/n, and shift in the dissipation, ΔD, for the third harmonic of the resonant frequency of quartz crystal sensors coated with S1, S4, S5, S6, H1, H4, H5 and H6 hydrogel films after addition of albumin or lysozyme; Figure S12: Antioxidant activity of resveratrol released from silicone hydrogels and their respective controls; Figure S13: Antioxidant activity of resveratrol released from HEMA hydrogels and their respective controls; Figure S14: Antioxidant activity of a 100 µg/mL resveratrol in ethanol:water 10:90 *v*/*v* solution, NaCl 0.9% and DPPH 0.1 mM solution.
