# Supplementary material for: Resveratrol-Loaded Hydrogel Contact Lenses with Antioxidant and Antibiofilm Performance
Source: Pharmaceutics. 2021 Apr 11;13(4):532. doi: 10.3390/pharmaceutics13040532 (PMC8069945; doi:10.3390/pharmaceutics13040532)
Supplement: Supplementary file 1 [file pharmaceutics-13-00532-s001.pdf]

pharmaceutics

Supplementary Materials: Resveratrol-Loaded Hydrogel Contact Lenses With Antioxidant and Antibiofilm Performance

María Vivero-Lopez, Andrea Muras, Diana Silva, Ana Paula Serro, Ana Otero, Angel Concheiro and Carmen Alvarez-Lorenzo

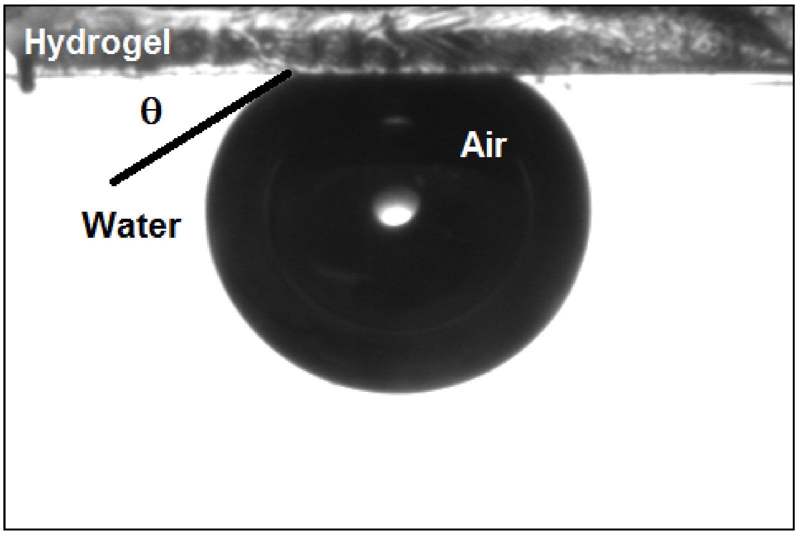

Figure S1. Image of a bubble adhered to a hydrogel contact lens when the captive bubble method was used for the measurement of the water contact angle (θ).

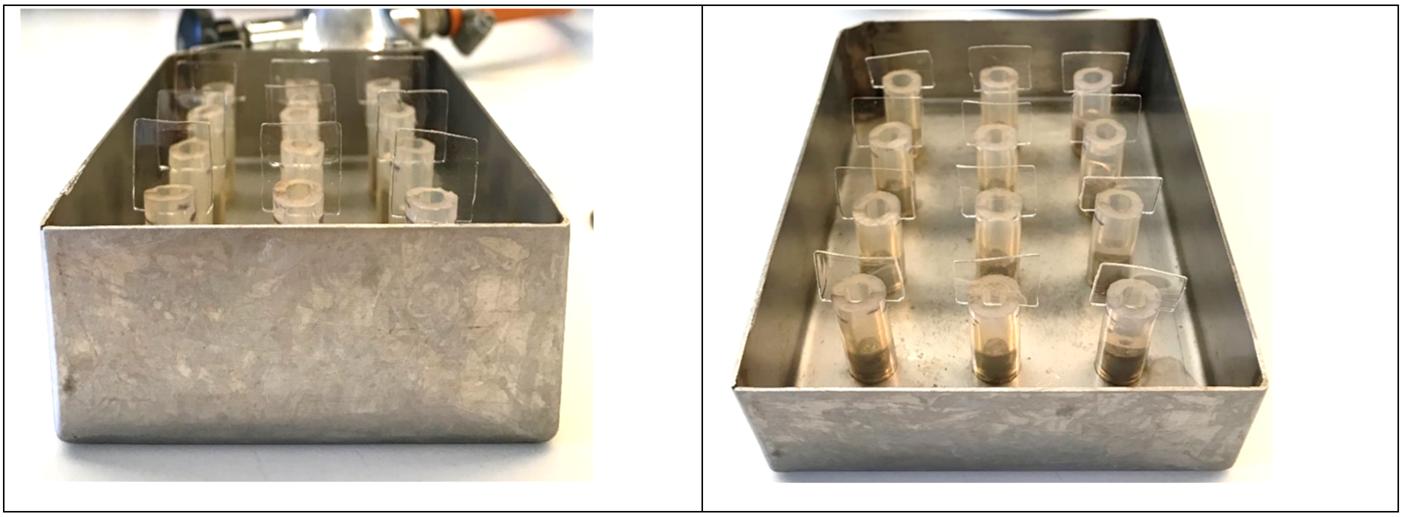

Figure S2. Amsterdam Active Attachment (AAA) model assembled with the tested hydrogels pieces.

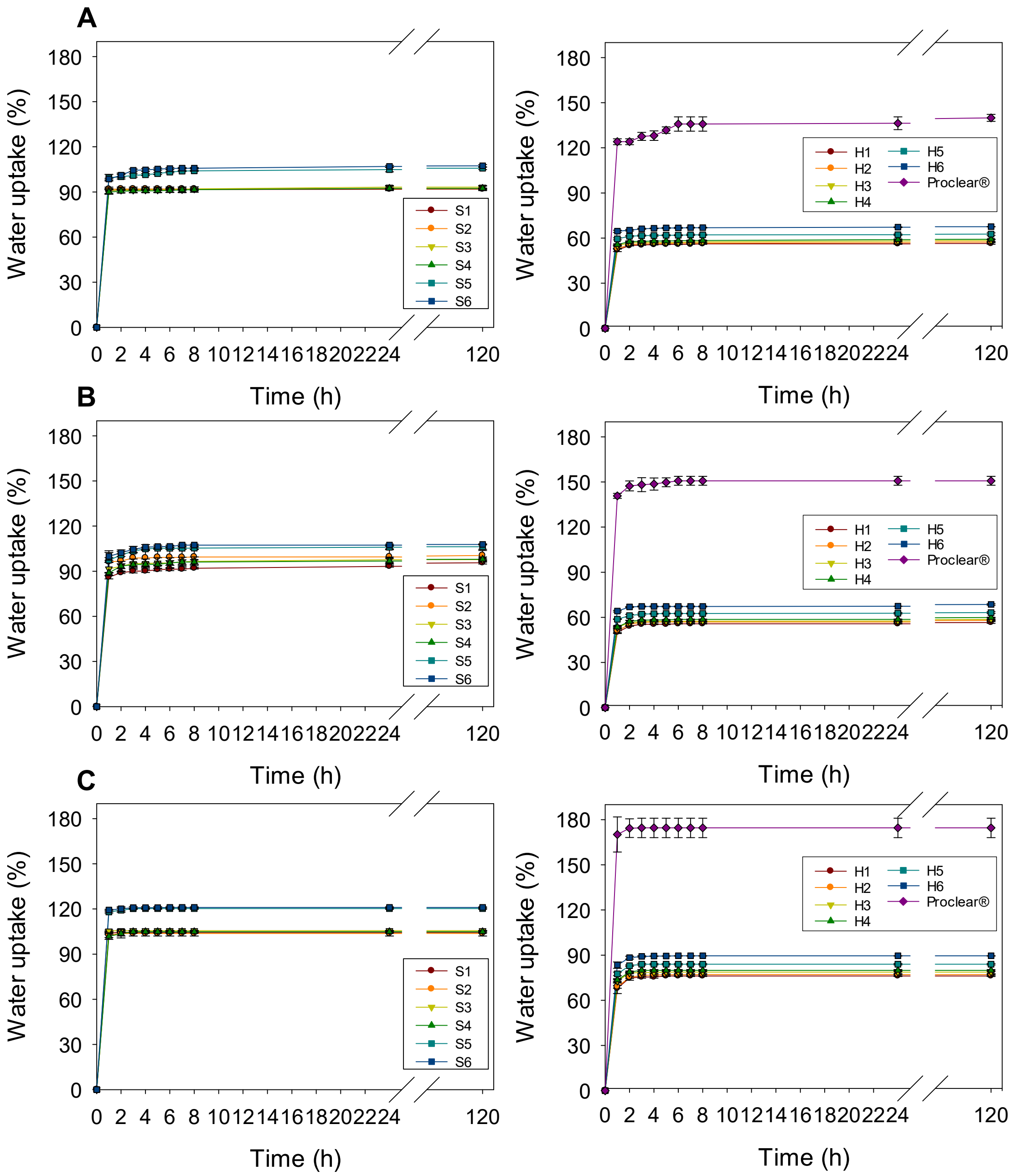

Figure S3. Water uptake (%) of silicone (left) and HEMA (right) hydrogels in water (**A**), SLF (**B**) and resveratrol loading solution (**C**). Codes as in Table 1.

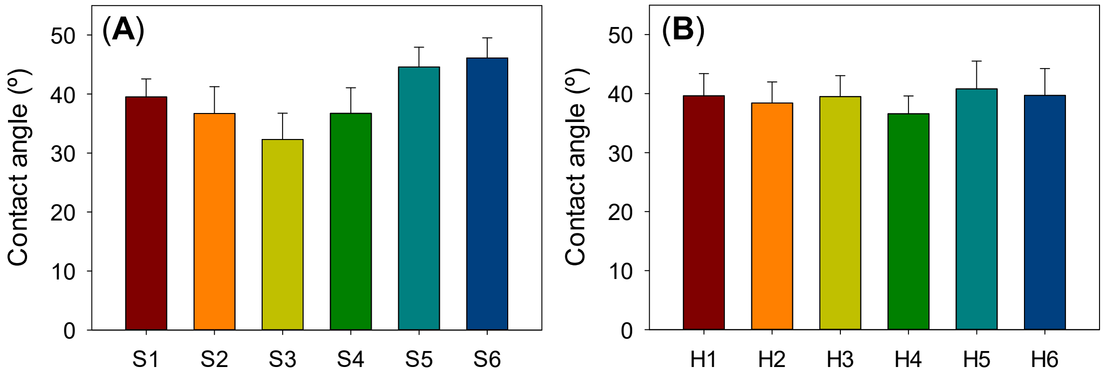

Figure S4. Water contact angles on the silicone-based hydrogels (**A**) and HEMA-based hydrogels (**B**). The error bars are ± the standard deviations.

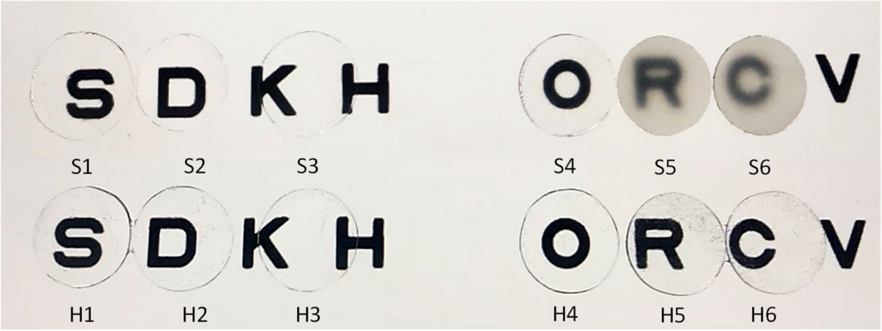

Figure S5. Reading test for silicone (**up**) and HEMA (**down**) hydrogels. Codes as in Table 1.

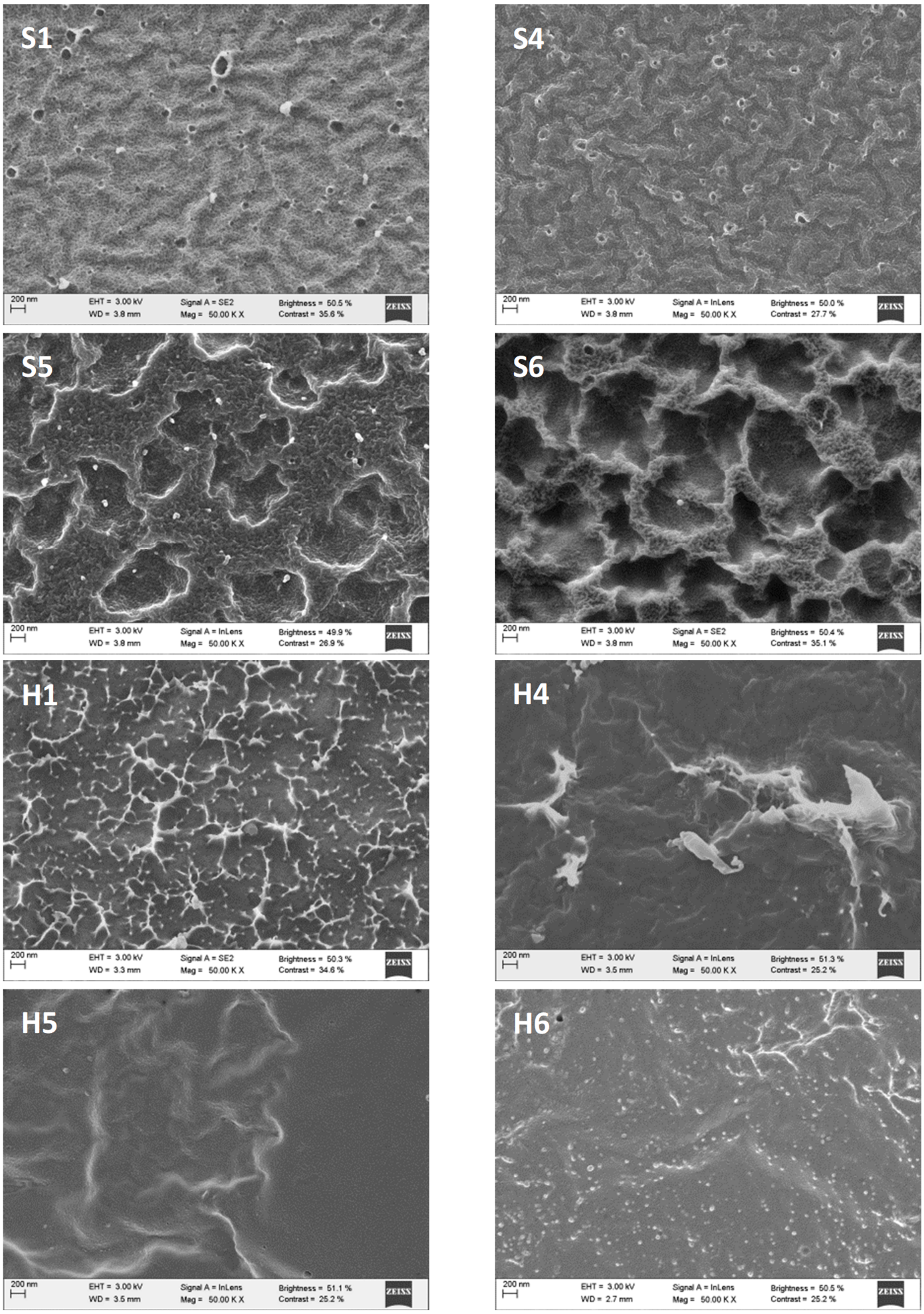

Figure S6. SEM images of freeze-dried hydrogels recorded at high magnification (50,000×). The scale bar corresponds to 200 nm. Codes as in Table 1.

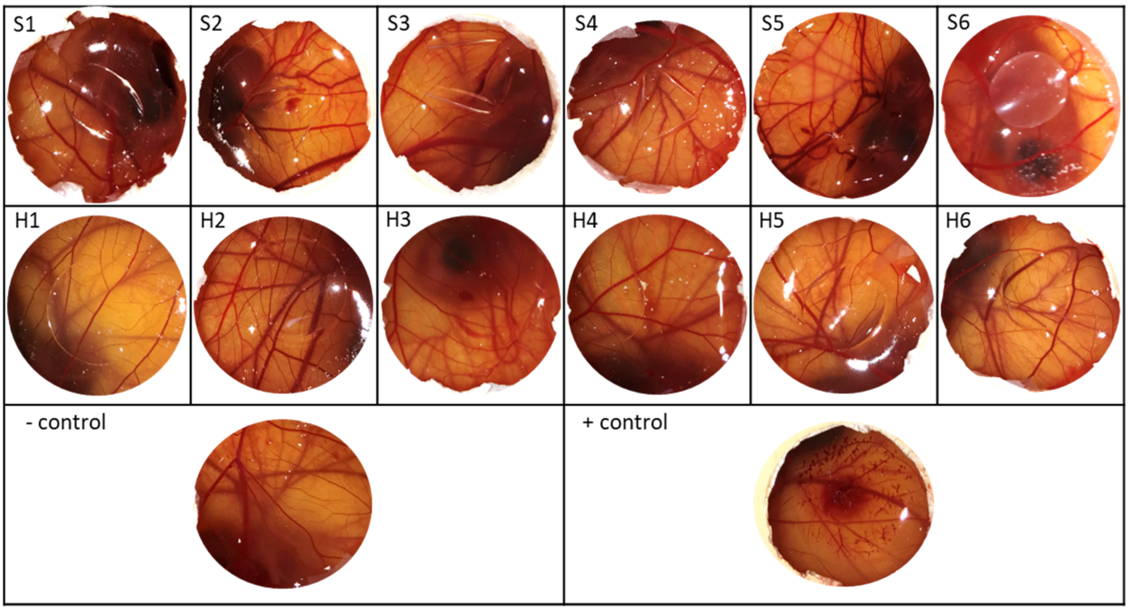

Figure S7. Images of the HET-CAM test. Codes as in Table 1.

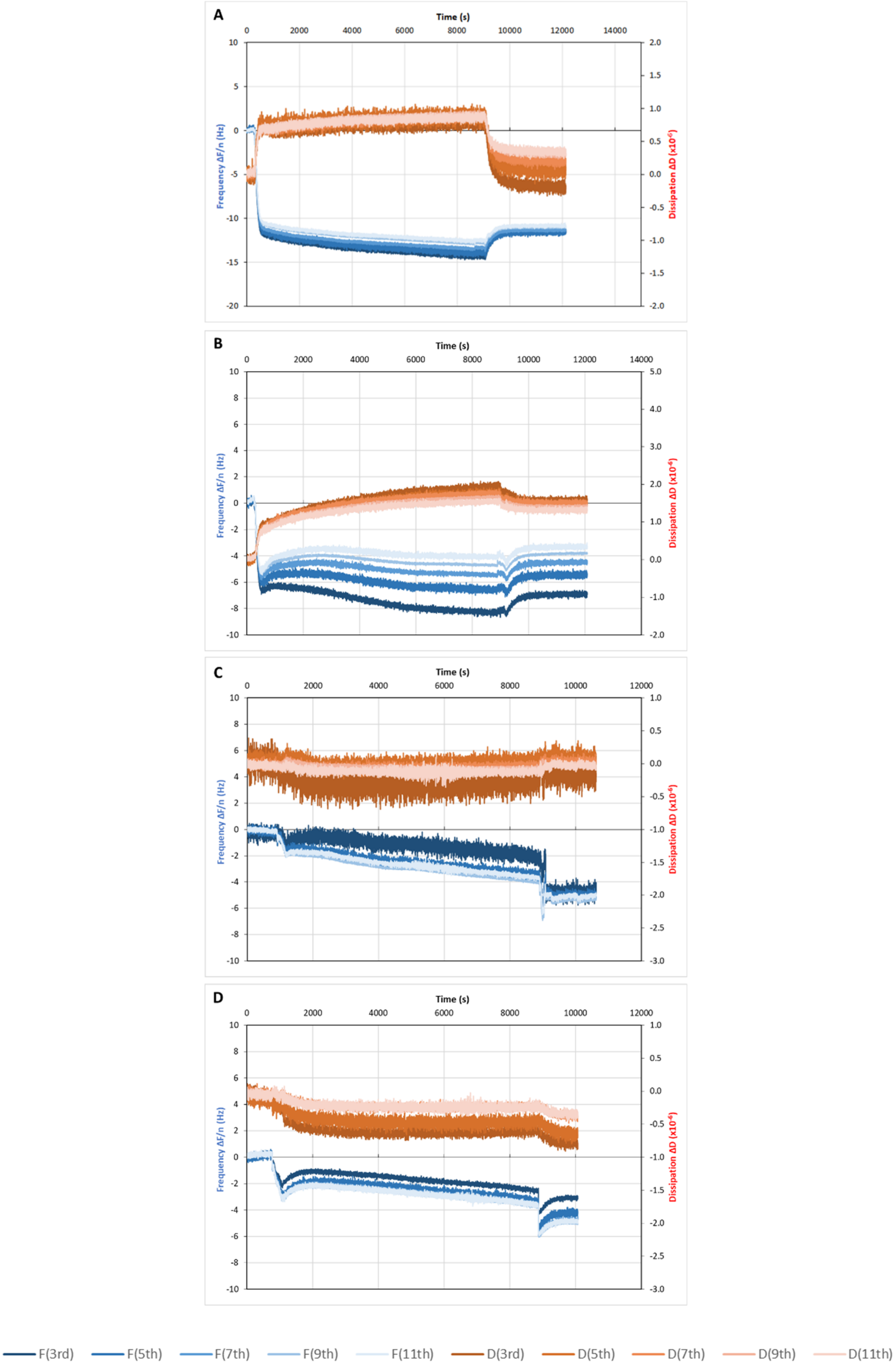

Figure S8. Normalized shift in the frequency, Δf/n, (blue line, left y-axis) and shift in the dissipation, ΔD, (red line, right y-axis) for the 3rd, 5th, 7th, 9th, and 11th harmonics of the resonant frequency of quartz crystal sensors coated with S1 (**A**), S4 (**B**), S5 (**C**) and S6 (**D**) hydrogel film. After few minutes equilibration in SLF, albumin solution was added (for ~150 min), and finally SLF rising solution was passed.

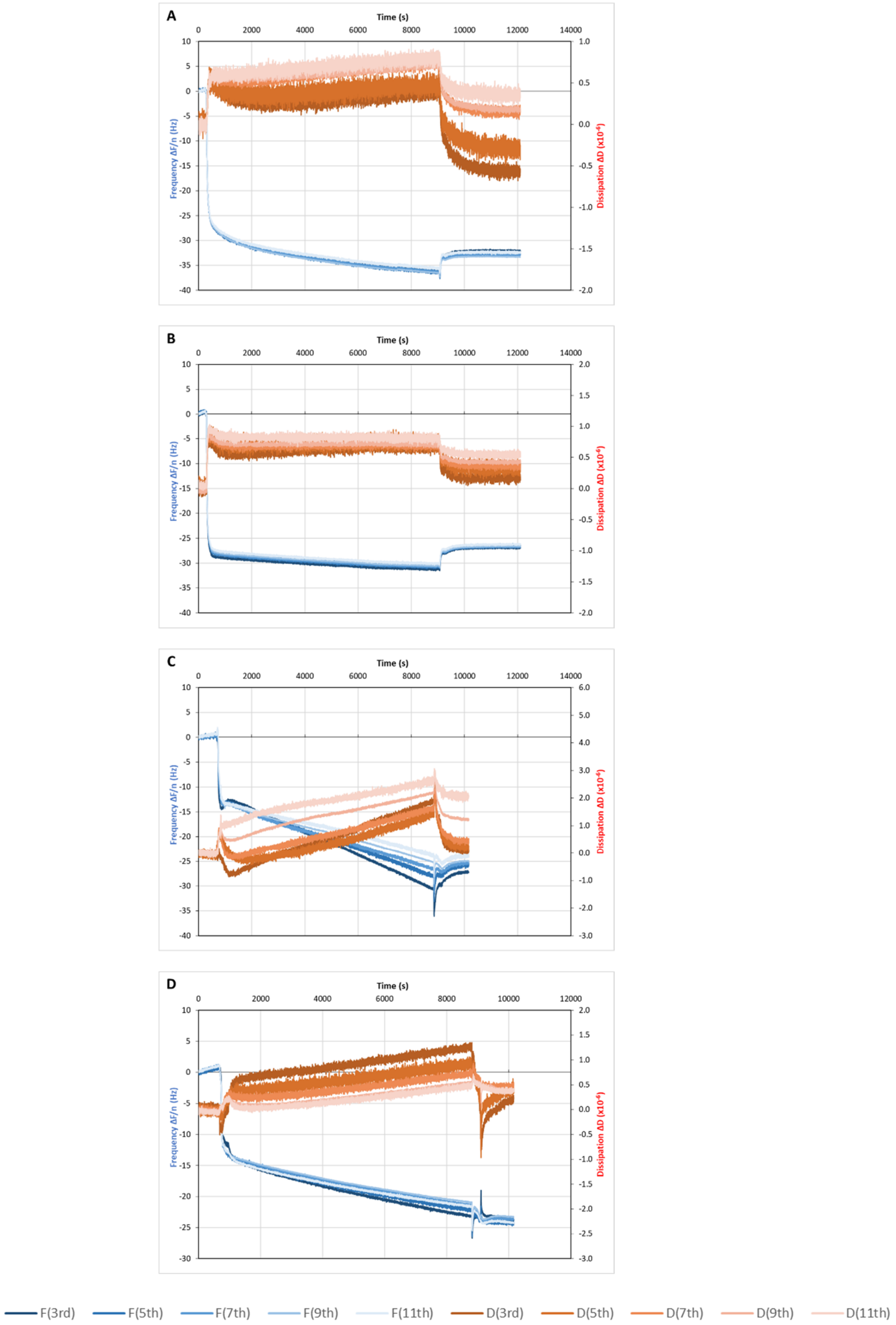

Figure S9. Normalized shift in the frequency, Δf/n, (blue line, left y-axis) and shift in the dissipation, ΔD, (red line, right y-axis) for 3rd, 5th, 7th, 9th, and 11th harmonics of the resonant frequency of quartz crystal sensors coated with S1 (**A**), S4 (**B**), S5 (**C**) and S6 (**D**) hydrogel film. After few minutes equilibration in SLF, lysozyme solution was added (for ~150 min), and finally SLF rising solution was passed.

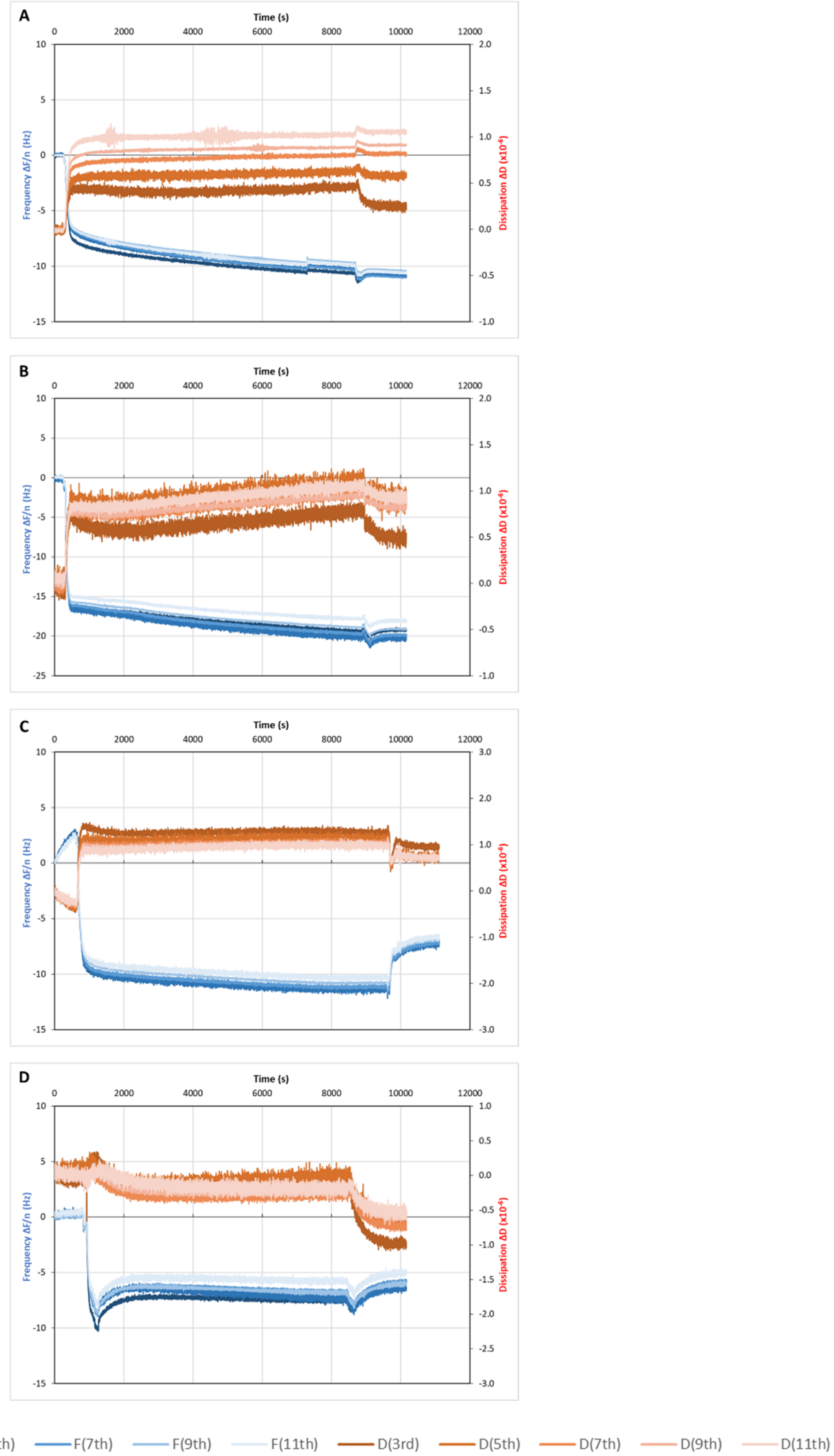

Figure S10. Normalized shift in the frequency, Δf/n, (blue line, left y-axis) and shift in the dissipation, ΔD, (red line, right y-axis) for the 3rd, 5th, 7th, 9th, and 11th harmonics of the resonant frequency of quartz crystal sensors coated with H1 (**A**), H4 (**B**), H5 (**C**) and H6 (**D**) hydrogel film. After few minutes equilibration in SLF, albumin solution was added (for ~150 min), and finally SLF rising solution was passed.

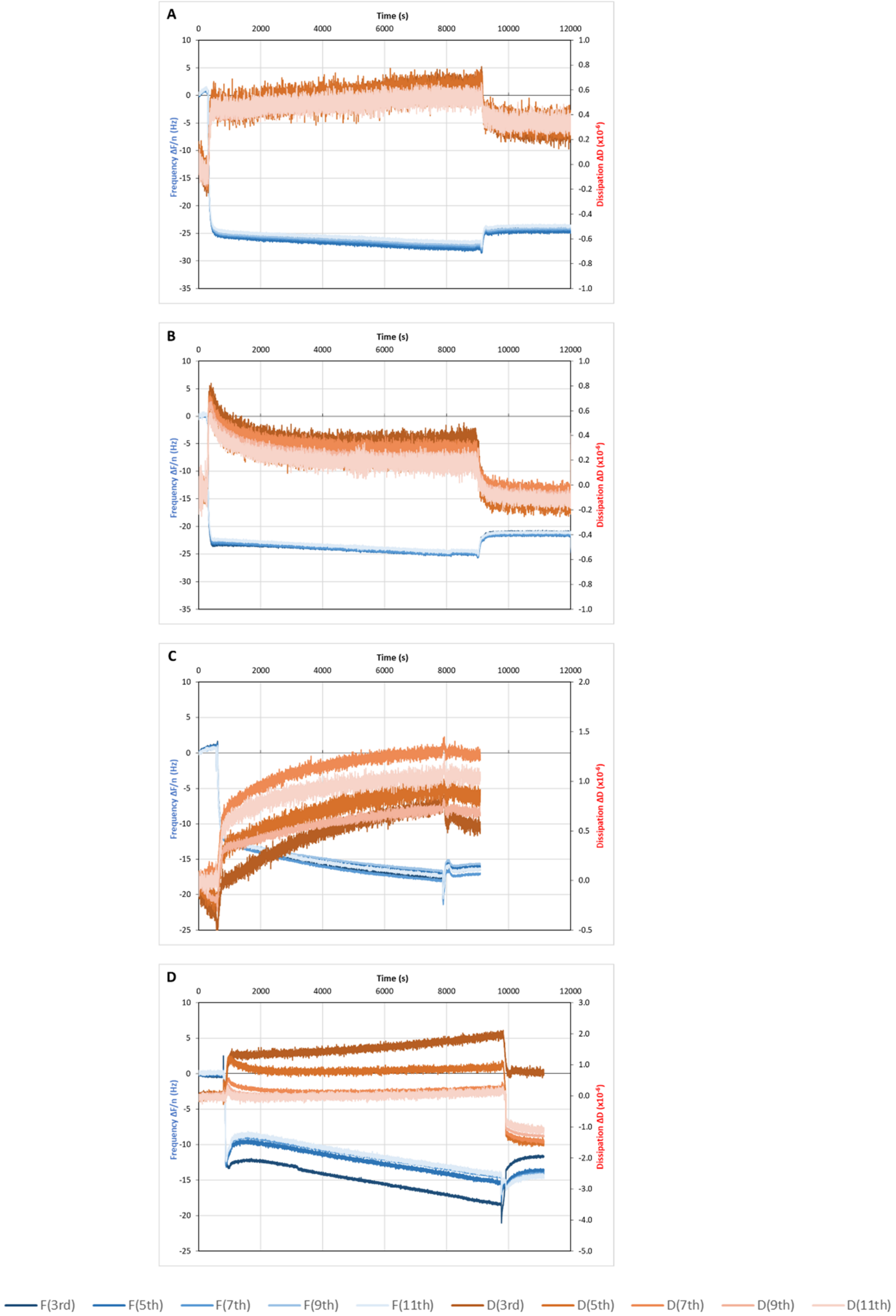

Figure S11. Normalized shift in the frequency, Δf/n, (blue line, left y-axis) and shift in the dissipation, ΔD, (red line, right y-axis) for the 3rd, 5th, 7th, 9th, and 11th harmonics of the resonant frequency of quartz crystal sensors coated with H1 (**A**), H4 (**B**), H5 (**C**) and H6 (**D**) hydrogel film. After few minutes equilibration in SLF, lysozyme solution was added (for ~150 min), and finally SLF rising solution was passed.

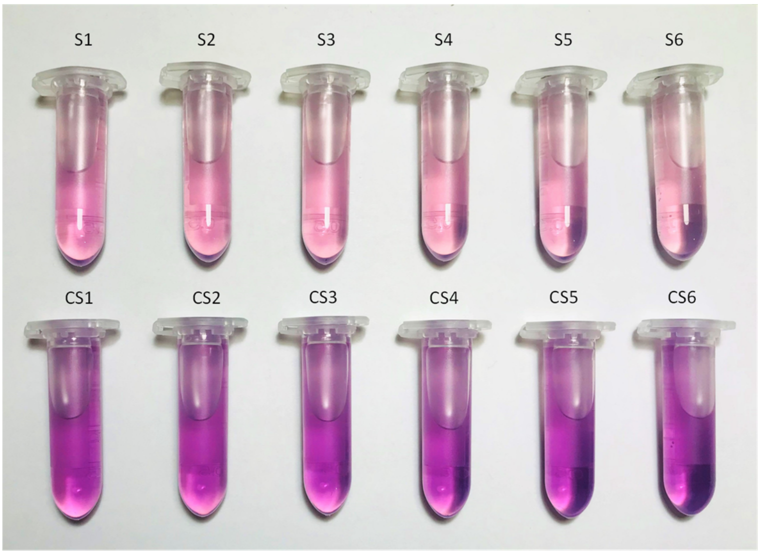

Figure S12. Antioxidant activity of resveratrol released from silicone-hydrogels (**up**) and their respective controls (**down**).

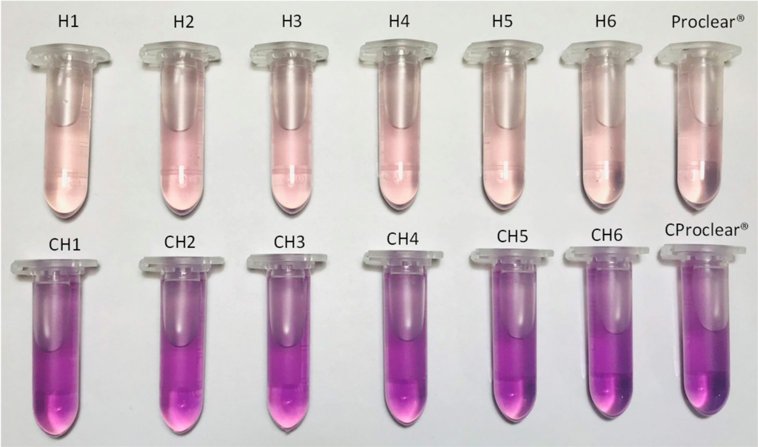

Figure S13. Antioxidant activity of resveratrol released from HEMA-hydrogels (**up**) and their respective controls (**down**).

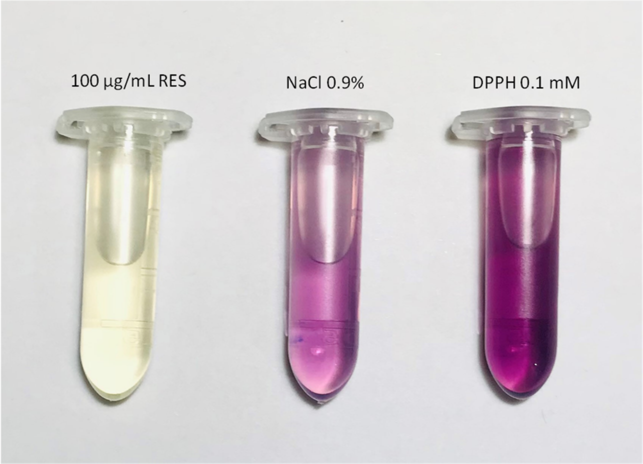

Figure S14. Antioxidant activity of a 100 µg/mL resveratrol solution in ethanol:water 10:90 *v*/*v* medium, a NaCl 0.9% solution, and a DPPH 0.1 mM solution.
